# Parallel regulatory circuits orchestrate biofilm formation in response to c-di-GMP levels and growth phase

**DOI:** 10.1371/journal.pgen.1011870

**Published:** 2025-09-15

**Authors:** Michael A. Trebino, Giordan Kitts, James R. J. Haycocks, Rachel Wheat, Issac Chaudry, Jin Hwan Park, Ivan Erill, David C. Grainger, Fitnat H. Yildiz

**Affiliations:** 1 Department of Microbiology and Environmental Toxicology, University of California, Santa Cruz, Santa Cruz, California, United States of America; 2 School of Biosciences, University of Birmingham, Birmingham, United Kingdom; 3 Center for Advanced Microbiome Research and Innovation, Institute for Genome Sciences, University of Maryland, School of Medicine, Baltimore, Maryland, United States of America; 4 Department of Biochemistry and Molecular Biology, University of Maryland, School of Medicine, Baltimore, Maryland, United States of America; 5 Department of Information and Communications Engineering, Universitat Autònoma de Barcelona, Bellaterra, Spain; 6 Department of Biological Sciences, University of Maryland, Baltimore County, Baltimore, Maryland, United States of America; Friedrich-Schiller-Universitat Jena, GERMANY

## Abstract

Biofilm formation is a highly regulated process that contributes to the environmental fitness of microorganisms, including pathogenic bacteria. The second messenger c-di-GMP is a critical regulator of biofilm formation whose cellular levels are tightly regulated by the abundance and activity of diguanylate cyclases (DGCs) and phosphodiesterases (PDEs). These enzymes synthesize and degrade c-di-GMP, respectively. The *Vibrio cholerae* VpvABC system encodes a DGC and is critical for biofilm formation; however, much remains unknown about its regulation. Here we demonstrate that the *vpvABC* system is transcriptionally regulated by c-di-GMP and the master biofilm regulators VpsT and VpsR. However, we also identify the alternative sigma factor RpoS as a positive regulator of *vpvABC*. RpoS is involved in the regulation of many c-di-GMP metabolism genes and plays a role in biofilm architecture, likely mediated in part through *vpvC*. In mature biofilms, *vpvA* transcription was highest near the biofilm substratum and VpsT, VpsR, and RpoS were critical for *vpvABC* transcription. Overall, our genetic dissection reveals the *vpvABC* system is regulated by two parallel circuits: a c-di-GMP sensing-circuit acting through VpsT and VpsR and a stationary growth phase circuit via RpoS. These findings underscore the multilayered regulatory mechanisms that precisely govern biofilm formation by a pathogen.

## Author summary

Biofilm formation enhances the environmental fitness of bacteria and relies on the second messenger c-di-GMP; the cellular levels of c-di-GMP are controlled by diguanylate cyclases (DGCs), which synthesize it, and phosphodiesterases (PDEs), which degrade it. However, much remains unknown about the transcriptional regulation of genes encoding DGCs and PDEs. This study reveals that the VpvABC c-di-GMP signaling system, which is critical for biofilm formation in *Vibrio cholerae*, is regulated by two key pathways: one driven by the biofilm regulators VpsT and VpsR, which respond to c-di-GMP levels, and another driven by the alternative sigma factor RpoS, which regulates gene expression during stationary phase and stress responses. These findings provide insight into the multifaceted regulation of pathogen biofilm formation through c-di-GMP-sensing and growth-phase-specific circuits.

## Introduction

Biofilms, comprising multicellular microbial communities encased in an extracellular matrix, are a common mode of bacterial growth [1]. The ability to form biofilms allows microorganisms to survive environmental fluctuations, increasing the likelihood of environmental fitness and success [2,3]. Indeed, biofilm formation is critical for the survival of *Vibrio cholerae* as it cycles between aquatic ecosystems and the human host, where this pathogen can cause cholera [4]. Biofilm formation is a highly regulated process involving multiple stages and different regulatory circuits [5–7]. The secondary messenger molecule cyclic di-guanosine monophosphate (c-di-GMP) is a major regulator of biofilm formation [8–10]. C-di-GMP signaling is widely conserved across bacteria, and intracellular levels of this second messenger are tightly regulated by diguanylate cyclases (DGCs), which carry GGDEF domains and synthesize c-di-GMP, and by phosphodiesterases (PDEs), which bear EAL or HD-GYP domains and degrade the molecule [8–10]. DGCs and PDEs typically contain sensing domains that allow bacteria to change their intracellular c-di-GMP pools in response to environmental signals [8–12]. Changes in intracellular c-di-GMP are sensed by c-di-GMP effector molecules, proteins, and RNAs, which bind to c-di-GMP and interact with a target component, thereby generating a molecular output [8,13–20]. The molecular functions of DGCs, PDEs and c-di-GMP effectors, and the integration of these systems into complex regulatory networks controlling biofilm formation, have recently begun to emerge.

*Vibrio cholerae*, the causative agent of the diarrheal disease cholera, forms biofilms by attaching to surfaces using pili, proteins, and other cell-surface appendages [21–24]. Once attached, *V. cholerae* cells produce extracellular matrix components, such as Vibrio polysaccharide (VPS) and matrix proteins, that help hold the cells together as microcolonies and mature biofilms [25–28]. In *V. cholerae,* c-di-GMP plays a crucial role in regulating the transition between the planktonic and biofilm lifestyles, biofilm matrix production, and biofilm dispersal [8,29–31]. While high levels of c-di-GMP promote biofilm formation, low levels promote biofilm dispersal and the planktonic lifestyle [8–10,18]. There are approximately 61 genes in the *V. cholerae* genome that encode DGCs and PDEs, and a number of these are critical for biofilm formation, motility, and virulence [30,32–36]. Changes in c-di-GMP are sensed by the transcriptional activators VpsT and VpsR, which positively regulate biofilm formation by directly activating transcription of biofilm matrix genes [14,37].

*V. cholerae* undergoes smooth-to-rugose phenotypic variation, which increases its evolutionary success and environmental fitness [38,39]. The rugose phenotype refers to the wrinkled appearance of the colony, which results from increased biofilm matrix production [40,41]. The rugose phenotype is often associated with high c-di-GMP levels and regulated by specific proteins of c-di-GMP signaling systems [32,38]. The VpvABC c-di-GMP signaling system is one such pathway and was previously identified as regulating the switch between two phenotypic variants of *V. cholerae* [38]. The VpvABC system is predicted to function as a three-component signaling module: VpvA is a porin, VpvB is a periplasmic binding protein, and VpvC is a membrane-bound diguanylate cyclase. VpvA likely imports an unknown signal, which is sensed by VpvB and VpvC, leading to activation of VpvC’s enzymatic activity. While VpvA and VpvB contribute to c-di-GMP levels and rugosity, their effects are less pronounced than those of VpvC [38]. Disruption of *vpvC* in the rugose variant produces smooth colonies and reduces the transcription of biofilm matrix genes [38]. The c-di-GMP binding transcription factor VpsT directly activates the *vpvABC* operon by binding to two sites in the upstream regulatory region [42]. Understanding how the *vpvABC* regulatory network is regulated is critical to determining when this system is activated in the *V. cholerae* life cycle, an important consideration for therapeutic targeting.

In this study, we characterized transcriptional regulation of the *vpvABC* operon in *V. cholerae*. Our data reveal that increases in intracellular c-di-GMP increase *vpvABC* expression via the transcriptional activators VpsT and VpsR. Moreover, we identify the alternative sigma factor RpoS as a positive regulator of *vpvABC*. As such, RpoS impacts intracellular c-di-GMP levels via *vpvC* expression. Furthermore, we show that RpoS regulates *vpvABC* independently of VpsT or VpsR. While RpoS controls many DGCs and PDEs, both directly and indirectly, only a few of these DGCs can restore rugosity in the absence of RpoS. The spatial profile of *vpvABC* expression and c-di-GMP production in mature biofilms provides further insight into how VpsT, VpsR and RpoS impact transcription of the *vpvABC* operon in a biofilm-relevant context. Together, our analysis establishes a regulatory model for *vpvABC* transcription and increases our understanding of the genetic and mechanistic regulation of this critical c-di-GMP signaling system in *V. cholerae*.

## Results

### Cellular c-di-GMP levels and the core biofilm transcriptional regulators VpsT and VpsR regulate the *vpvABC* operon

**Fig 1:**
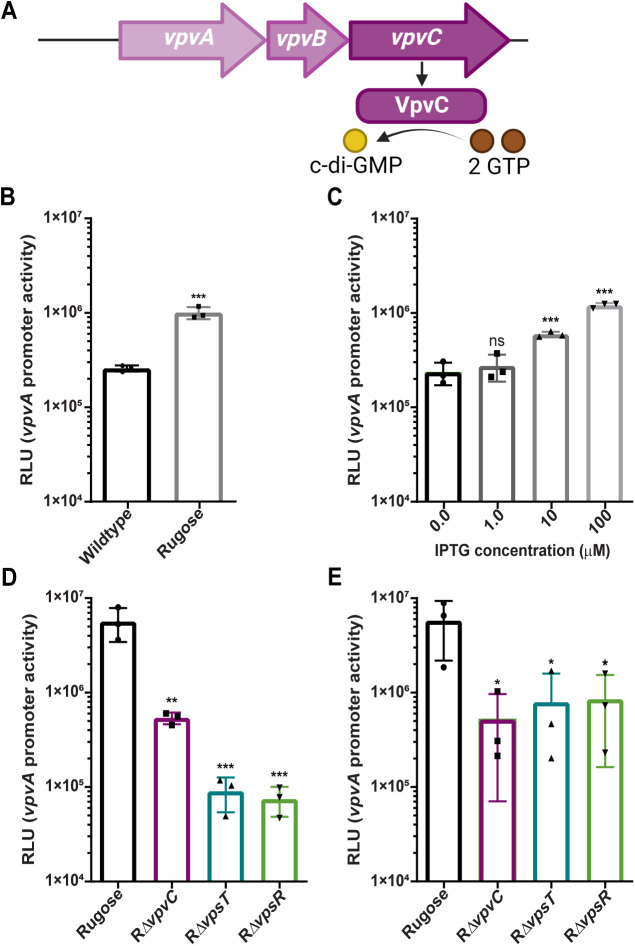
The *vpvABC* operon is regulated by c-di-GMP signaling, VpsT, and VpsR. (A) Genetic organization of the *vpvABC* operon. (B) Bar graph showing means and standard deviations of P*vpvA-luxCDABE* promoter activity in the wild-type and rugose variant strains based on three independent biological replicates grown in liquid media. Relative light units (RLU) are reported in luminescence counts per minute per milliliter per OD600. The rugose variant was compared to wild-type using an unpaired two-tailed t-test. *** = P < 0.001. (C) Bar graph showing means and standard deviations of P*vpvA-luxCDABE* promoter activity in wild-type::Ptac-VCA0956. Strains were induced with 0.0, 1.0, 10, and 100 µM IPTG at the time of inoculation in liquid media. (D and E) Bar graph showing means and standard deviations of P*vpvA-luxCDABE* promoter activity in the rugose variant (R), RΔ*vpvC*, RΔ*vpsT*, and RΔ*vpsR* during the exponential and stationary growth phases in liquid media, respectively. Means were compared to uninduced (B) or the rugose variant (C and D) with a one-way ANOVA followed by Dunnett’s test. * = P < 0.05, ** = P < 0.01, *** = P < 0.001, and **** = P < 0.0001.

To dissect the transcriptional regulation of the *vpvABC* operon, we constructed a luminescence-based transcriptional reporter, P*vpvA*-*luxCDABE* (P*vpvA-lux*) (Fig 1A). We quantified the promoter activity of *vpvABC* by measuring luminescence in *V. cholerae* A1552 wild-type and the rugose variant during exponential and stationary growth. Transcription of *vpvABC* was approximately 4-fold higher in the rugose variant compared to wild-type (Fig 1B). As the main difference between the two strains is elevated c-di-GMP due to a single nucleotide polymorphism in *vpvC*, we sought to determine if increasing c-di-GMP can impact *vpvABC* expression. Using a wild-type *V. cholerae* A1552 strain harboring an inducible DGC (Ptac-VCA0956), we measured P*vpvA*-*lux* promoter activity in the presence of varying IPTG concentrations. We observed a dose-dependent increase in promoter activity at 10 and 100 µM IPTG (Fig 1C). We also analyzed P*vpvA-lux* promoter activity in rugose cells lacking *vpvC*, referred to as RΔ*vpvC*, as this gene is the main driver of the increased c-di-GMP in the rugose variant. Deletion of *vpvC* led to a marked decrease in transcription during both the exponential and stationary growth phases (Fig 1D and 1E). This suggests that elevated intracellular c-di-GMP levels lead to increased *vpvABC* transcription.

VpsT and VpsR are c-di-GMP-dependent transcriptional regulators [14,37,43]. Since increased c-di-GMP resulted in increased *vpvABC* transcription, we measured P*vpvA-lux* promoter activity in the rugose variant, RΔ*vpsT*, and RΔ*vpsR* strains. Absence of either VpsT or VpsR during the exponential growth phase resulted in decreased *vpvABC* transcription (Fig 1D). In the stationary phase, loss of VpsT and VpsR had a reduced impact on *vpvABC* expression, indicating their primary role as activators during exponential growth where c-di-GMP levels are highest (Fig 1E). Collectively, these findings show that *vpvABC* transcription is positively regulated by increased cellular c-di-GMP levels and by the VpsT and VpsR transcriptional regulators, with the latter predominantly acting during exponential growth.

### RpoS regulates *vpvABC* transcription

**Fig 2:**
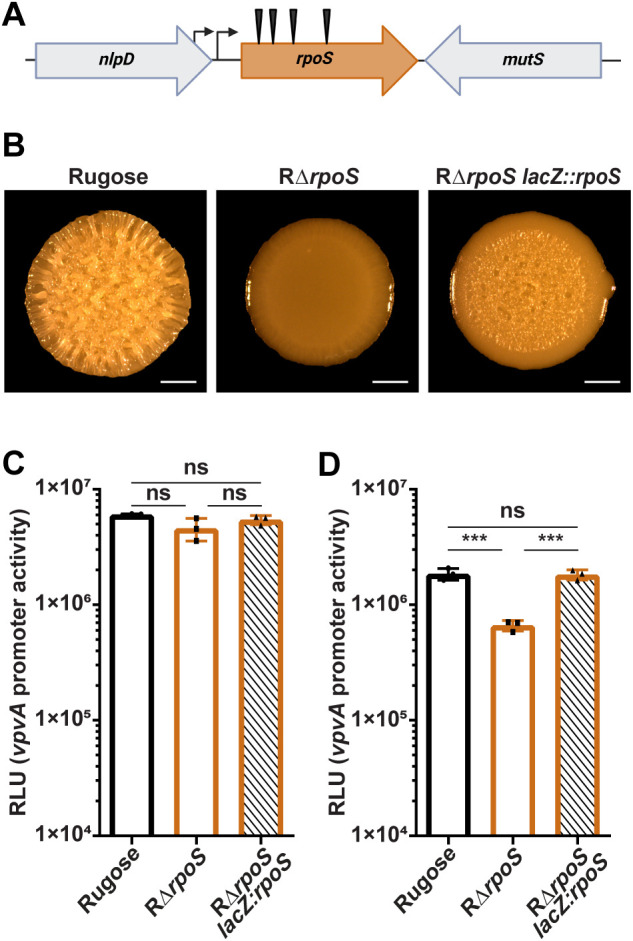
RpoS regulates transcription of the *vpvABC* system. (A) Representation of mTn10 transposon insertion sites in *rpoS*. (B) Representative images of colony morphologies of the rugose variant, RΔ*rpoS*, and RΔ*rpoS lacZ::rpoS* colony biofilms grown on solid media agar plates for 48 hours at 30°C. (C and D) Bar graph showing means and standard deviations of P*vpvA*-*luxCDABE* promoter activity from three biological replicates in the rugose variant, RΔ*rpoS*, and RΔ*rpoS lacZ::rpoS* during the exponential and stationary growth phases in liquid media, respectively. Means were compared using a one-way ANOVA followed by Tukey’s test. *** = P < 0.001.

To identify additional regulators of the *vpvABC* system, we generated a mTn10 transposon mutant library in the rugose genetic background. We used the *V. cholerae* A1552 rugose variant as *vpvABC* expression is higher in this genetic background compared to the wild-type. To screen the transposon library, we constructed a P*vpvA-sfGFP* transcriptional reporter and introduced this reporter to the mTn10 library en masse. We then screened for mutants with altered fluorescence compared to the rugose variant (S2 Table). We screened 12,000 transposon mutants on solid media, identified 76 mutants with increased or decreased fluorescence, and mapped the transposon insertion sites to 17 different genes (S2 Table). As expected, mutations in *vpsT*, *vpsR*, and *vpvA* diminished fluorescence (S1 Fig). We found that insertions in the gene encoding the alternative sigma factor *rpoS* (VC0534) significantly reduced fluorescence (S1 Fig). Four unique insertion sites were identified, all producing the same phenotype (Fig 2A).

To follow up on this finding, we next generated a strain lacking RpoS in the rugose genetic background (RΔ*rpoS*) and found that these cells had a smooth colony phenotype when grown as colony biofilms on solid media (Fig 2B), indicating that biofilm matrix production is decreased. Introducing a wild-type copy of *rpoS* into the RΔ*rpoS* strain, at the neutral *lacZ* site, under the control of its two upstream promoters, partially restored rugosity. This suggests that RpoS regulates the rugose phenotype. It was previously shown that transcriptional readthrough of VC0533, the gene upstream of *rpoS*, contributes to *rpoS* transcription in *V. cholerae*; this would explain the partial complementation we observe [44,45]. We measured P*vpvA-lux* promoter activity in RΔ*rpoS* and RΔ*rpoS lacZ::rpoS* cells in exponential and stationary growth phases. The lack of RpoS did not impact *vpvABC* transcription during exponential growth (Fig 2C). However, during stationary phase, lack of RpoS significantly decreased *vpvABC* transcription, while *vpvABC* expression was restored to levels similar to the rugose variant in the RΔ*rpoS lacZ::rpoS* strain (Fig 2C and 2D). These findings indicate that RpoS does not transcriptionally regulate the *vpvABC* system during the exponential phase of bacterial growth but exerts a regulatory effect during the stationary phase. This growth phase-dependent regulation aligns with the established expression profile of RpoS which is known to be upregulated during the transition to stationary phase and under various stress conditions [44,46–48].

### RssB represses *vpvABC* transcription and rugosity

**Fig 3:**
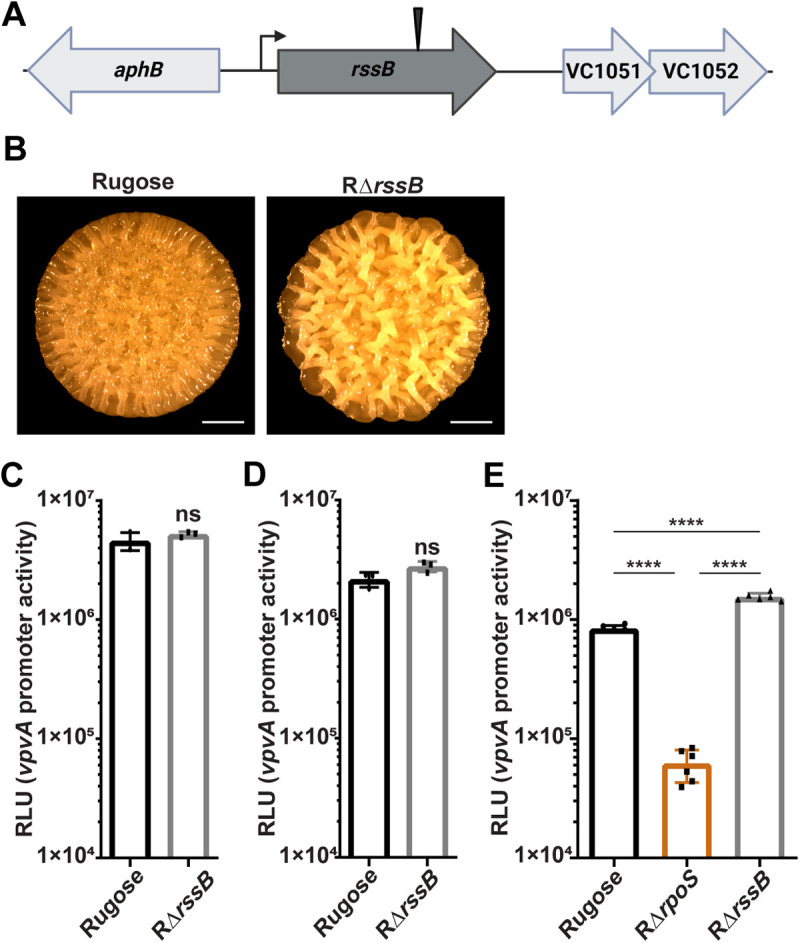
RssB regulates the *vpvABC* system. (A) Schematic showing mTn10 transposon insertion sites in *rssB*. (B) Representative images of colony morphologies of the rugose variant and RΔ*rssB* grown as colony biofilms on solid media agar plates for 48 hours at 30 °C. (C and D) Bar graph of means and standard deviations of P*vpvA-luxCDABE* promoter activity in the rugose variant and RΔ*rssB* during the exponential and stationary growth phases in liquid media, respectively. Unpaired two-tailed t-test was used to compare the rugose variant and RΔ*rssB*. (E) Bar graph of means and standard deviations of P*vpvA-luxCDABE* promoter activity in the rugose variant and RΔ*rssB* from colony biofilms grown for 48 hours on solid media agar plates at 30°C using six biological replicates. Means were compared using a one-way ANOVA followed by Tukey’s test. **** = P < 0.0001.

An insertion in VC1050 (*rssB*), a gene encoding the adaptor protein that directs RpoS for proteolysis, was identified in our transposon mutagenesis screen and led to elevated fluorescence (S1 Fig) [49,50]. This increase in fluorescence is likely due to accumulation of RpoS resulting from impaired proteolytic degradation. Only a single mutant with this phenotype was recovered from the screen (Fig 3A).

To confirm that RssB functions in *vpvABC* regulation, we generated a strain lacking RssB in the rugose variant. When grown as colony biofilms on solid media, the RΔ*rssB* strain appeared more opaque and exhibited elevated corrugation ridges compared to the rugose parent strain, a phenotype linked to increased matrix synthesis and enhanced biofilm-forming ability (Fig 3B). We next measured P*vpvA-lux* promoter activity in RΔ*rssB* in exponential and stationary growth phases.

While loss of *rssB* caused a slight increase in *vpvABC* transcriptional activity, the difference was not statistically significant compared to the rugose variant under these conditions, showing a fold change of 1.13 during exponential growth phase and 1.28 during stationary growth phase (Fig 3C and 3D). Since the transposon screen was performed on colony biofilms grown on nutrient agar plates, we measured P*vpvA-lux* promoter activity in the rugose variant and RΔ*rssB* on solid media grown as colony biofilms. Under these conditions, *vpvABC* transcription was significantly increased in RΔ*rssB* with a fold change of 1.84, suggesting that RssB may impact RpoS stability more robustly in biofilm-grown cells (Fig 3E). Consistent with our findings from the transposon screen, *vpvABC* transcriptional activity was significantly decreased in RΔ*rpoS* compared to both the rugose variant and RΔ*rssB* (Fig 3E).

### RpoS regulates cellular c-di-GMP levels in *V. cholerae*

**Fig 4:**
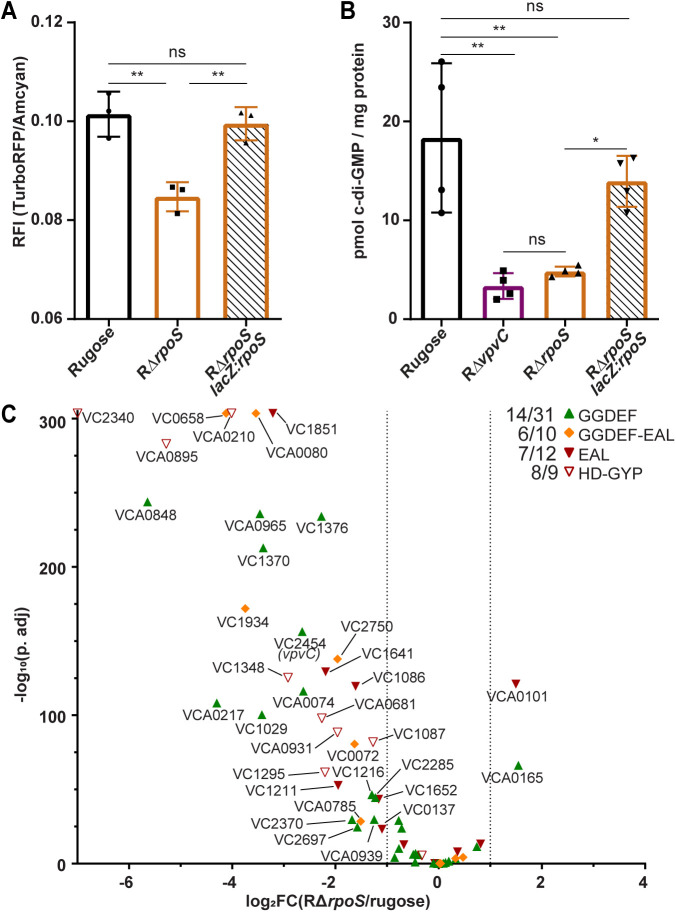
RpoS controls intracellular c-di-GMP levels and regulates transcription of c-di-GMP metabolism genes. (A) Bar graph showing means and standard deviations of c-di-GMP in the rugose variant, RΔ*rpoS*, and RΔ*rpoS lacZ::rpoS* from three independent biological replicates grown in liquid media. Relative fluorescent intensity (RFI) was calculated from the ratio of fluorescence intensity of TurboRFP to Amcyan. (B) Bar graph showing means and standard deviations of c-di-GMP in the rugose variant, RΔ*vpvC*, RΔ*rpoS*, and RΔ*rpoS lacZ::rpoS* from four independent biological replicates grown in liquid media. c-di-GMP was quantified via LC MS/MS and reported in pmols of c-di-GMP per milligram of total protein. Means were compared using a one-way ANOVA followed by Tukey’s test. * = P < 0.05 and ** = P < 0.01. (C) Volcano plot of differentially regulated c-di-GMP metabolism genes in RΔ*rpoS* compared to the rugose variant. Genes with GGDEF domains are represented by green upward triangles, GGDEF-EAL domains by orange diamonds, EAL domains by red downward triangles, and HD-GYP domains by red empty downward triangles. Dotted lines indicate a log_2_FC threshold of -1 and 1.

Since VpvC impacts cellular c-di-GMP levels, we sought to determine if RpoS also shifts cellular c-di-GMP levels. We measured cellular c-di-GMP levels using two complementary approaches, a c-di-GMP-specific riboswitch fluorescent reporter and LC-MS/MS [36,51]. In this dual fluorescent reporter, TurboRFP production is regulated by two c-di-GMP-binding riboswitches and thus reports upon cellular c-di-GMP levels. We also introduced an AAV degron to TurboRFP to allow for more refined spatial measurement of c-di-GMP levels. The fluorescent protein AmCyan encoded in this biosensor is produced constitutively and is used for normalization. The relative fluorescence intensity (RFI) of cells that express this reporter is directly proportional to c-di-GMP levels. Using the c-di-GMP specific riboswitch fluorescent reporter harboring an AAV degron, significantly lower c-di-GMP levels were found in RΔ*rpoS* compared to the rugose variant using both assays (Fig 4A and 4B). However, this phenotype was not observed in RΔ*rpoS lacZ::rpoS*. Therefore, RpoS impacts cellular c-di-GMP levels.

To determine if RpoS controls expression of genes encoding DGCs and PDEs in *V. cholerae*, we performed transcriptional profiling on colony biofilms grown on solid media from the rugose variant and RΔ*rpoS*. Using criteria of a corrected p-value (p.adj.) <= 0.05 and a fold change of 2 or greater in transcript abundance, 14.88% of the *V. cholerae* transcriptome was differentially regulated in RΔ*rpoS* when compared to the rugose variant. Of these differentially expressed genes, 349 were down-regulated and 180 were up-regulated (S2 Fig).

Based on the link to *vpvC*, we focused on the role of RpoS in c-di-GMP metabolism and found that genes involved in biofilm formation and c-di-GMP metabolism were down-regulated. Indeed, when RΔ*rpoS* was compared to the rugose variant, 33 out of 61 c-di-GMP metabolism genes were down-regulated while 2 were up-regulated. This suggests that RpoS is a major regulator of c-di-GMP signaling in *V. cholerae* beyond *vpvC* (Fig 4C).

### Colony rugosity is induced by RpoS in a VpvC-dependent manner

To determine if any of these DGCs contribute to colony rugosity phenotypes, we analyzed colony corrugation in our collection of *V. cholerae* strains harboring single DGC deletions on solid media grown as colony biofilms (S3 Fig). We found that none of the single deletions, except *vpvC*, phenocopied RΔ*rpoS.* Consistent with previous observations, deletion of two DGCs (VC1376 and VC2285, *cdgM* and *cdgL,* respectively) reduced colony corrugation, while deletion of the PDE VCA0785 (*cdgC*) increased colony corrugation [52]. This finding suggests that under the conditions tested, RpoS regulates rugosity through transcriptional regulation of *vpvABC*.

**Fig 5:**
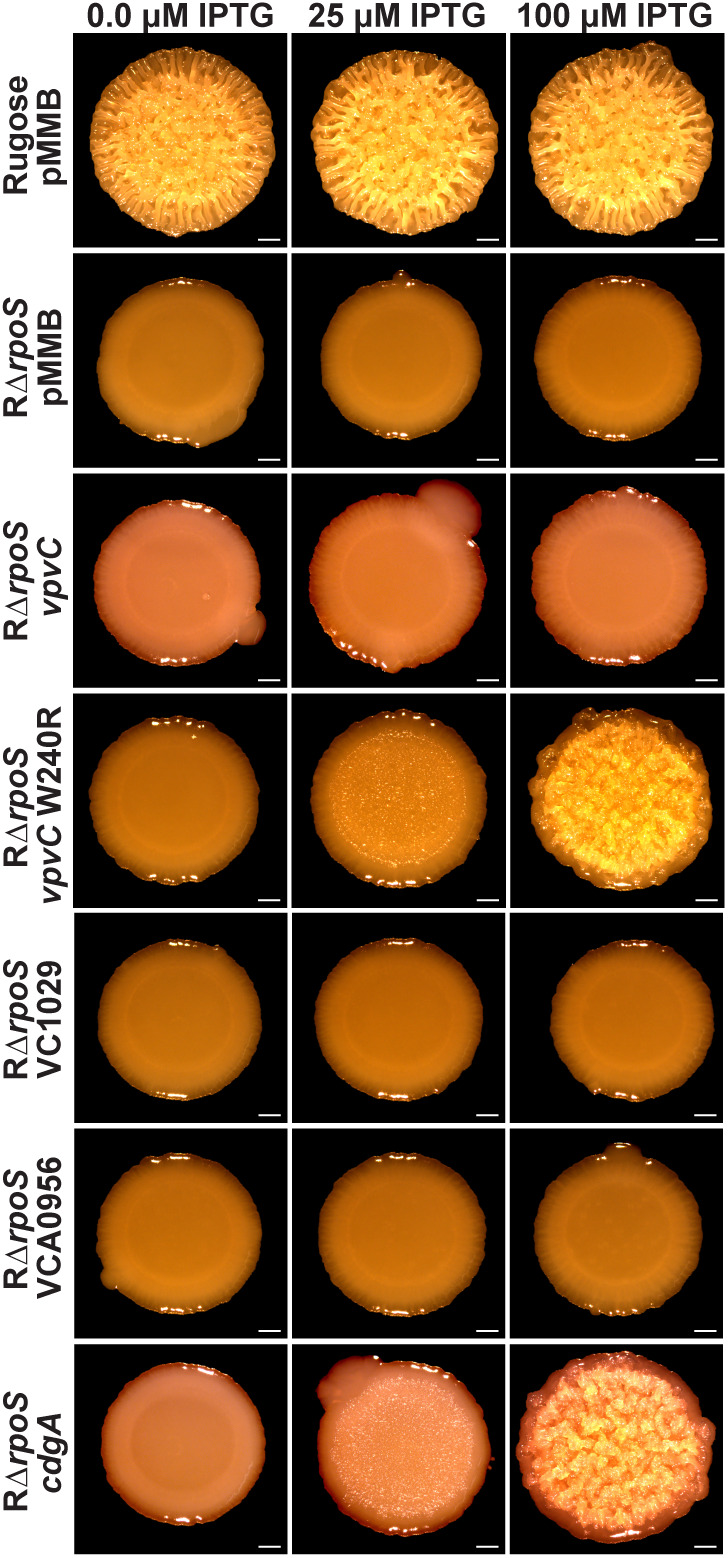
RpoS controls rugosity via VpvC and CdgA. Representative images of colony morphologies of rugose pMMB, RΔ*rpoS* pMMB, RΔ*rpoS* pMMB-*vpvC*, RΔ*rpoS* pMMB-*vpvC* W240R, RΔ*rpoS* pMMB-VC1029, RΔ*rpoS* pMMB-VCA0956, and RΔ*rpoS* pMMB-*cdgA*. Colony biofilms were grown for 72 hours on solid media agar plates at 30°C, and DGC induction was achieved using 0.0, 25, and 100 µM IPTG.

To further investigate this premise, we also overexpressed five different DGCs in RΔ*rpoS* and analyzed colony corrugation as a cellular readout of c-di-GMP levels. We compared strains carrying a wild-type copy of *vpvC*, *vpvC* W240R (the rugose variant mutation), VC1029, which is directly regulated by VpsT, VCA0956 (a DGC that has been extensively used to modulate cellular c-di-GMP levels), and *cdgA* (VCA0074, a DGC that can increase *vpvABC* expression when overexpressed). Overexpression of VCA0956 leads to increased intracellular c-di-GMP levels, as we previously demonstrated using multiple detection methods [18,24,30]. Overexpression of VpvC (W240R), but not the wild-type protein, partially restored the rugose phenotype at 25 µM IPTG in the absence of *rpoS* (Fig 5). In contrast, overexpression of VC1029, and VCA0956 at 100 µM did not alter colony corrugation (Fig 5). Overexpression of CdgA at 25 µM IPTG, like VpvC (W240R), began to restore rugosity in the absence of *rpoS* (Fig 5). This suggests that, in our rugose variant, RpoS contributes to c-di-GMP and biofilm formation through both *vpvC* and *cdgA*. Since rugosity is not fully abolished in cells lacking *cdgA,* RpoS must primarily drive rugosity through control of *vpvC* (S3 Fig). Consistent with previous studies, this suggests that specific modes of rugosity are driven by particular players in the c-di-GMP network rather than by overall changes in global c-di-GMP levels [30,35,53,54].

### RpoS directly binds to the promoter of *vpvABC*

We know that many DGCs and PDEs are regulated by elevated c-di-GMP levels through VpsT and VpsR [42,53,55]. Since RpoS positively regulates many of these DGCs and PDEs, as evidenced by RNA-seq of the rugose variant, we sought to determine if this regulation also occurs in a wild-type background. We found that 28 of 61 c-di-GMP metabolizing genes were downregulated and 2 were upregulated in Δ*rpoS* compared to the wild-type strain (S4 Fig). Of the DGCs/PDEs that are differentially regulated in the wild-type dataset, 85.7% are also differentially regulated in the rugose dataset. This overlap is 84.8% when considering only downregulated genes. However, the magnitude of differential expression of the DGCs/PDEs was generally reduced in the wild-type strain. These results suggest that while RpoS regulates the expression of c-di-GMP signaling genes, the overall magnitude and significance of these changes are modulated by additional factors, including c-di-GMP levels.

**Fig 6:**
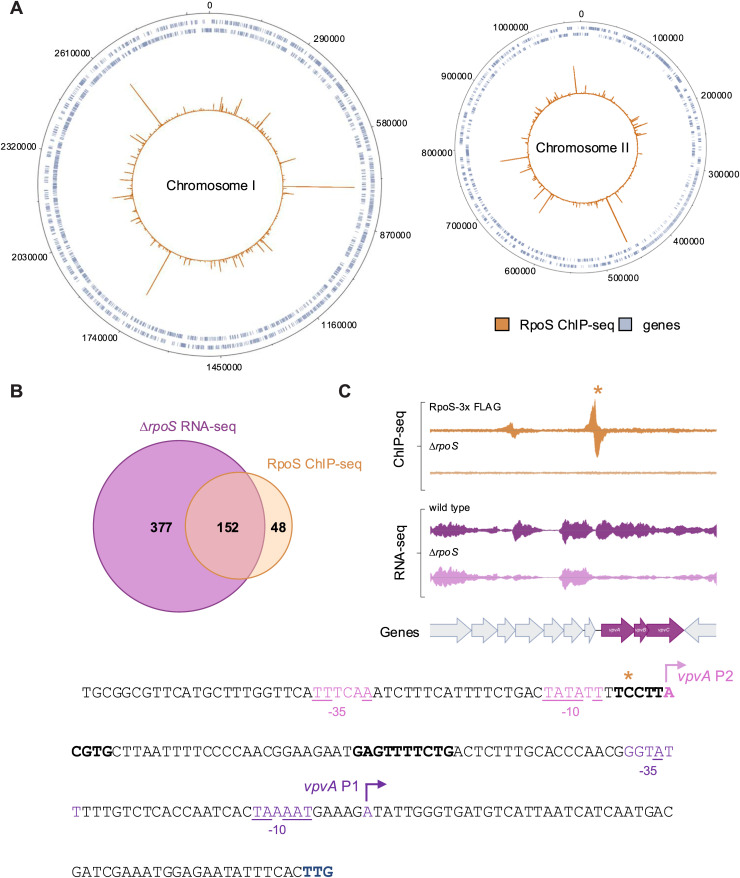
RpoS regulates *vpvABC* through direct binding. (A) Binding of RpoS across both *V. cholerae* chromosomes. In each plot, the outer two tracks (blue) are genes orientated in the forward or reverse direction. The RpoS ChIP-seq binding signal (orange) is the inner profile. Chromosome coordinates are labeled. (B) Venn diagram showing overlap between genes differentially expressed in the absence of RpoS and RpoS-binding sites identified by ChIP-seq. (C) Transcription of the *vpvABC* operon during stationary phase requires RpoS. ChIP-seq data for RpoS binding (top) and RNA-seq data for RpoS-dependent transcription (bottom) mapped onto the *vpvABC* operon. Genes are shown as block arrows. The sequence of the *vpvABC* regulatory region is shown below, with the *vpvA* start codon shown in blue. P1 promoter elements are shown in purple, and P2 promoter elements shown in pink. In both cases, bases matching the consensus -35 and -10 sequences are underlined, and the transcription start site is indicated by a bent arrow. VpsT binding sites are shown in bold. The center of the RpoS ChIP-seq peak is indicated by an asterisk above the sequence.

Whether this manifests downstream through c-di-GMP receptors or directly through RpoS DNA binding remains to be determined. We next sought to determine if RpoS directly binds to the *vpvABC* promoter and whether it binds to the promoters of other DGCs and PDEs. To do so, we used ChIP-seq to map the binding of RpoS across the *V. cholerae* genome (Fig 6A). This analysis was performed in a wild-type background with a C-terminal 3xFLAG-tagged RpoS inserted at its endogenous locus. RpoS binding mapped to 200 genes and 152 of these genes overlapped with genes identified in our RNA-seq dataset (Fig 6B). We identified 19 c-di-GMP signaling-associated genes with RpoS binding peaks, and 12 of these were significantly downregulated according to RNA-seq. There was a prominent RpoS binding peak upstream of the *vpvABC* operon, consistent with the loss of downstream RNA-seq reads in Δ*rpoS* cells (Fig 6C). The *vpvABC* operon has two promoters, P1 and P2, with two VpsT binding sites at P1 but no known binding sites at P2 [42]. Notably, the RpoS peak near *vpvA* mapped to P2. This indicates that *vpvABC* regulation can occur at P1 via c-di-GMP sensing through VpsT or at P2 through RpoS.

### VpsT, VpsR, and RpoS impact *vpvABC* transcription in mature biofilms

To examine the biological relevance of our findings, we next tested whether VpsT, VpsR, and RpoS regulation of *vpvABC* occurs during biofilm formation. Thus, we constructed a fluorescent reporter where the *vpvABC* upstream regulatory region drives the expression of *sfGFP* harboring an AAV degron. This reporter also carries a constitutively transcribed mCardinal, used for normalization. Relative gene expression was thus calculated as the ratio of sfGFP fluorescence normalized to mCardinal fluorescence. We analyzed the *vpvA* spatial expression pattern in mature biofilms of the rugose variant, RΔ*vpvC*, RΔ*vpsT*, RΔ*vpsR*, and RΔ*rpoS* strains grown under flow conditions in a microfluidic chamber.

**Fig 7:**
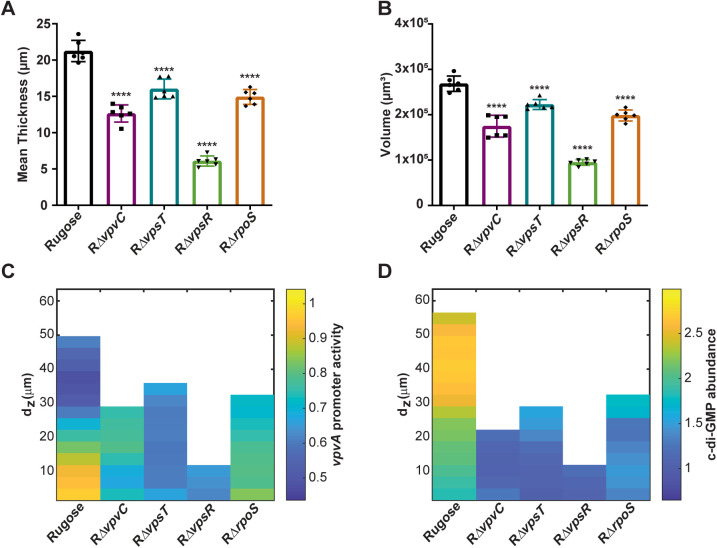
VpsT, VpsR, and RpoS regulate *vpvABC* expression and c-di-GMP levels in biofilms. (A and B) Bar graph showing means and standard deviations for thickness and volume, respectively. Data are shown for the rugose variant, RΔ*vpvC*, RΔ*vpsT*, RΔ*vpsR*, and RΔ*rpoS* strains from three technical replicates of two independent biological replicates grown in flow cell chambers. (C) Kymograph of *vpvA* promoter activity based on P*vpvA-sfGFP* expression in the rugose variant, RΔ*vpvC*, RΔ*vpsT*, RΔ*vpsR*, and RΔ*rpoS*, in mature biofilms grown in flow cell chambers. Biofilm depth is reported in µm and relative vpvABC expression as a ratio of sfGFP intensity/mCardinal intensity. (D) Kymograph of c-di-GMP abundance (RFI) in the rugose variant, RΔ*vpvC*, RΔ*vpsT*, RΔ*vpsR*, and RΔ*rpoS* in mature biofilms using the same images from panels A and B.

The biofilm-forming ability of these mutant strains was reduced compared to the rugose parental strain (Fig 7A and 7B). Lack of any of the above genes led to a decrease in mean biofilm thickness (Fig 7A). RΔ*vpvC*, RΔ*vpsT*, and RΔ*rpoS* had a mean biofilm thickness of 12–16 µm with the rugose variant having a mean thickness of 21 µm. RΔ*vpsR* was only able to form monolayers with a mean thickness of 6 µm (Fig 7A). Lack of these genes also led to a decrease in biofilm volume compared to the rugose variant (Fig 7B). In the context of the biofilm, *vpvABC* reporter transcription in the rugose variant was highest near the substratum (Fig 7C). This transcription pattern was abolished in RΔ*vpsT* and RΔ*vpsR* and was markedly reduced in RΔ*vpvC* and RΔ*rpoS* (Fig 7C). This suggests that the *vpvABC* regulators VpsT, VpsR, and RpoS control *vpvABC* expression in a mature biofilm.

As VpvC contributes to cellular c-di-GMP levels in planktonically grown cells, we sought to evaluate c-di-GMP levels in biofilms using the c-di-GMP-specific riboswitch fluorescent reporter under the same conditions where we analyzed *vpvABC* transcription. In the rugose variant, c-di-GMP levels varied as a function of biofilm thickness, i.e., highest at the periphery of the biofilm (Fig 7D). In RΔ*vpvC*, RΔ*vpsT*, and RΔ*vpsR* c-di-GMP abundance was markedly decreased compared to the rugose variant (Fig 7D). In RΔ*rpoS*, c-di-GMP abundance was reduced, but was higher than RΔ*vpvC*, RΔ*vpsT*, and RΔ*vpsR* (Fig 7D). Collectively these findings suggest that VpsT, VpsR, and RpoS regulate spatial transcription of *vpvC* in the rugose variant. While cellular c-di-GMP levels contribute to *vpvABC* transcription in biofilms, *vpvABC* expression does not overlap with the regions of highest c-di-GMP abundance. Indeed, c-di-GMP accumulation in RΔ*vpvC*, RΔ*vpsT*, R*ΔvpsR*, and RΔ*rpoS* was markedly reduced in areas close to the substratum. Our data demonstrate that c-di-GMP signaling circuitries controlled by the systems impacted in these mutant strains contribute to c-di-GMP levels in mature biofilms.

### RpoS, VpsT, and VpsR regulate *vpvABC* through parallel pathways

**Fig 8:**
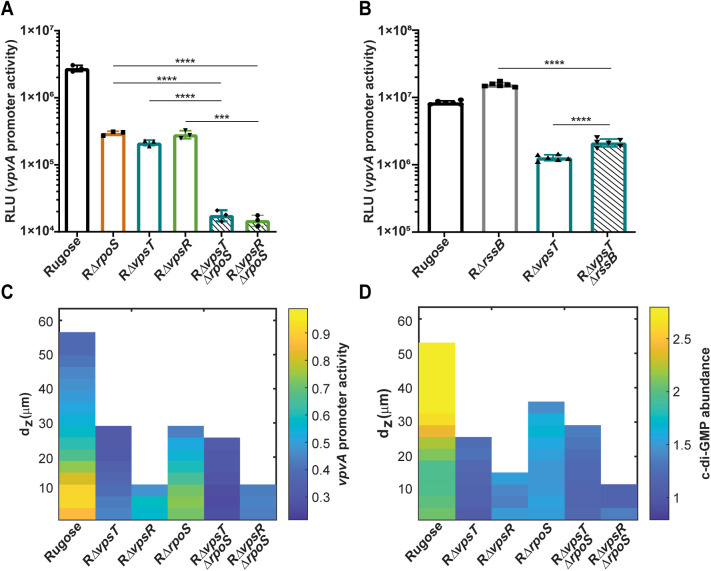
RpoS regulates *vpvABC* expression through a distinct pathway from VpsT and VpsR. (A) Bar graph showing means and standard deviations of P*vpvA*-*luxCDABE* expression in the rugose variant, RΔ*vpsT*, RΔ*vpsR*, RΔ*rpoS*, RΔ*vpsT*Δ*rpoS*, and RΔ*vpsR*Δ*rpoS* from three independent biological replicates grown in flow cell chambers. Strains were compared using an unpaired two-tailed t-test. *** = P < 0.001 and **** = P < 0.0001. (B) Bar graph showing means and standard deviations of P*vpvA*-*luxCDABE* expression in the rugose variant, RΔ*rssB*, RΔ*vpsT*, and RΔ*vpsT*Δ*rssB* from colony biofilms grown for 48 hours on solid media agar plates at 30 °C using six biological replicates. Strains were compared using an unpaired two-tailed t-test. **** = P < 0.0001. (C) Kymograph of *vpvA* promoter activity based on P*vpvA*-*sfGFP* expression in the rugose variant, RΔ*vpsT*, RΔ*vpsR*, RΔ*rpoS*, RΔ*vpsT*Δ*rpoS*, and RΔ*vpsR*Δ*rpoS* in 24 hour mature biofilms from three technical replicates of two independent biological replicates grown in flow cell chambers. (D) Kymograph of c-di-GMP abundance (RFI) in the rugose variant, RΔ*vpsT*, RΔ*vpsR*, RΔ*rpoS*, RΔ*vpsT*Δ*rpoS*, and RΔ*vpsR*Δ*rpoS* in 24 hour mature biofilms.

To determine if RpoS regulates the *vpvABC* system through the same pathway as VpsT and VpsR, we deleted *rpoS* in the RΔ*vpsT* and RΔ*vpsR* mutant strains. We measured *vpvA* promoter activity during the stationary growth phase for the rugose variant, RΔ*vpsT*, RΔ*vpsR*, RΔ*rpoS*, RΔ*vpsT*Δ*rpoS* and RΔ*vpsR*Δ*rpoS* strains. Transcription of *vpvA* was significantly decreased in RΔ*vpsT*Δ*rpoS* and RΔ*vpsR*Δ*rpoS* compared to RΔ*vpsT*, RΔ*vpsR*, and RΔ*rpoS* (Fig 8A). To determine if increased RpoS levels could rescue *vpvABC* transcription in the absence of *vpsT*, we measured *vpvA* promoter activity in RΔ*rssB, R*Δ*vpsT, and* RΔ*vpsT*Δ*rssB* colony biofilms, as our data above indicated that the *rssB* mutant impacts surface-grown cells. Lack of RssB in RΔ*vpsT* led to a small but significant increase in *vpvA* promoter activity (Fig 8B). These results suggest that RpoS regulates *vpvABC* expression through a parallel pathway to VpsT and VpsR.

To determine if this regulatory hierarchy exists during biofilm formation, we measured *vpvABC* transcriptional activity in mature biofilms. We observed a marked decrease in *vpvABC* transcription in RΔ*vpsT*Δ*rpoS* and RΔ*vpsR*Δ*rpoS* compared to RΔ*vpsT*, RΔ*vpsR*, and RΔ*rpoS* (Fig 8C). This finding is consistent with RpoS acting through a parallel regulatory pathway to that of VpsT and VpsR. We also analyzed c-di-GMP accumulation in mature biofilms in these sets of strains. Overall, the c-di-GMP abundance pattern in RΔ*vpsT*Δ*rpoS* phenocopies that of RΔ*vpsT* (Fig 8D). However, there is a decrease in c-di-GMP abundance in the RΔ*vpsR*Δ*rpoS* mutant compared to RΔ*vpsR* and RΔ*rpoS* (Fig 8D).

Collectively these findings suggest that RpoS regulates *vpvABC* in a pathway parallel to that of VpsT and VpsR. Furthermore, these three regulators control the abundance of c-di-GMP in mature biofilms suggesting that they regulate transcription of c-di–GMP signaling circuitries activated in mature biofilms.

### Phylogenetic distribution of the *vpvABC* operon

To further characterize the *vpvABC* operon and its importance for adaptation in *V. cholerae*, we performed a comparative genomics analysis of this operon distribution in the *Vibrio* genus and among Pseudomonadota reference genomes, measuring the structural and sequence conservation of the *vpvABC* operon through reciprocal BLAST and superimposing the findings on a RecA phylogeny (S5 Fig). Our results reveal that the *vpvABC* operon is conserved in many freshwater and marine *Vibrio* species, predominantly pathogenic or associated with marine invertebrates, but partially or completely absent in several non-pathogenic marine *Vibrio* species, which predominantly contain only *vpvC* homologs. This variable distribution and the substantial divergence in sequence identity for all three operon genes suggest that *vpvABC* is ancestral to the *Vibrio* genus and that it is particularly adaptive for pathogenic and animal-associated species. Outside *Vibrio*, we find fully conserved instances of the *vpvABC* operon in several marine and freshwater species belonging to the Gammaproteobacteria or Betaproteobacteria genera, such as *Grimontia*, *Halopseudomonas*, *Shewanella*, *Undibacterium* or *Aquincola*. A few bacterial species from both classes contain homologs of all three *vpvABC* operon genes, but only *vpvA* and *vpvB* form an operon. Conservation of the *vpvAB* operon configuration, in the absence of *vpvC*, is also detected in a large number of Gammaproteobacteria or Betaproteobacteria species. These include predominantly water-associated genera, such as *Shewanella*, *Alteromonas*, *Duganella* or *Roseateles*, but also some land-associated genera, like *Xanthomonas*, *Janthinobacterium* or *Thauera*. The conservation, in the absence of *vpvC*, of the *vpvAB* two-gene operon among a diverse group of bacteria suggests that these two genes may form a conserved sensing unit that became later linked to the *vpvC* diguanylate cyclase.

## Discussion

C-di-GMP signaling governs the transition from planktonic to biofilm lifestyles [8]. Yet which of the many DGCs and PDEs feed specific regulatory circuits remains unclear. One way to generate c-di-GMP signaling specificity is to produce and activate DGCs and PDEs within defined circuits and in response to specific signals [53]. *V. cholerae* has 30 DGCs, 11 hybrid DGC/PDEs, and 20 PDEs, and we are only beginning to understand the molecular mechanisms of their specificity [34]. Among these, we focused our study on the VpvABC c-di-GMP signaling system. VpvABC forms a three-component signal sensing system whose output is known to control c-di-GMP levels. Here, we uncovered parallel regulatory pathways that control the *vpvABC*-encoded diguanylate cyclase through distinct promoters to modulate biofilm formation.

We found that the exponential growth phase transcription of the *vpvABC* c-di-GMP signaling system is regulated by the c-di-GMP-binding transcriptional activators VpsT and VpsR. Our finding is consistent with VpsT ChIP-seq studies, which found two binding sites upstream of the *vpvA* transcriptional start site, including P1, and revealed that VpsT directly regulates *vpvA* transcription [42]. VpsR likely influences *vpvA* transcription through direct VpsR-mediated regulation of *vpsT* [55]. While VpvC generated c-di-GMP contributes to increased *vpvABC* transcription, we found that c-di-GMP-dependent regulation of *vpvABC* is not restricted to c-di-GMP produced by VpvC, as overexpression of VCA0956 was able to induce *vpvA* expression.

We identified RpoS as a direct regulator of *vpvABC* transcription during stationary growth. In *V. cholerae*, VpsT and c-di-GMP repress RpoS transcription [56]. Our findings suggest that during exponential growth, VpsT and c-di-GMP activate *vpvABC* transcription while repressing *rpoS* expression. In stationary phase, declining c-di-GMP levels reduce VpsT activity, relieving this repression. As a result, RpoS can accumulate and activate *vpvABC* via the P2 promoter.

**Fig 9:**
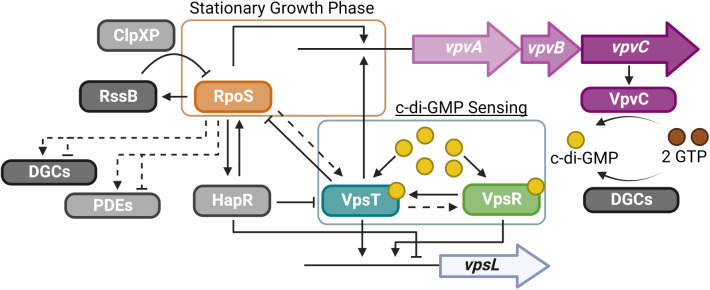
The VpvABC system is controlled by two distinct regulatory circuits. Illustration of the regulatory model of *vpvABC* operon. The c-di-GMP sensing circuit utilizes VpsT and VpsR to sense increases in c-di-GMP which is produced by VpvC and other DGCs. VpsT, when bound to c-di-GMP, will directly bind to the *vpvA* promoter and activate transcription. VpsR likely activates *vpsT* expression which will enhance *vpvABC* expression downstream. The stationary growth phase circuit utilizes RpoS to activate *vpvABC* transcription as well as influence transcription of additional DGCs and PDEs. While the mechanism underlying RpoS-mediated regulation of *vpvA* is unclear, RpoS levels can influence *vpvA* promoter activity and subsequently impact global c-di-GMP levels. RssB can recognize RpoS and recruit the protease ClpXP under appropriate conditions to degrade RpoS which subsequently influences *vpvABC* transcription. This allows for c-di-GMP-independent expression of the operon.

The RpoS regulatory network is highly complex and involves transcriptional regulation, translational regulation, regulation of binding affinity, and protein stability [49,50,56–59]. One specific regulator of RpoS is RssB, a proteolytic targeting factor. RssB binds to RpoS, thus allowing ClpXP to bind and degrade RpoS [49,50]. We observed that loss of RssB led to increased *vpvA* promoter activity, likely due to RpoS accumulation in the absence of ClpXP-mediated proteolysis of RpoS. Interestingly, this was enhanced in surface-grown cells and led to an increase in colony corrugation. We also observed that differences in *vpvA* promoter activity between RΔ*rpoS* and the rugose variant were greater in cells cultured on a surface compared to those in suspension. Collectively, we identified two distinct regulatory circuits for the *vpvABC* system: a circuit utilizing VpsT and VpsR as c-di-GMP sensors and a stationary growth phase circuit involving RpoS (Fig 9).

Having established the distinct contributions of VpsT/VpsR and RpoS to *vpvABC* expression during exponential and stationary phases, we next examined how these regulators shape *vpvABC* expression and c-di-GMP distribution in mature biofilms. We found that areas of the biofilm with the highest *vpvABC* expression did not necessarily correspond to highest levels of c-di-GMP. The apparent decoupling between *vpvABC* promoter activity and c-di-GMP levels may reflect distinct growth states within the biofilm. At the periphery, high c-di-GMP levels and DGC expression drive VpsT and VpsR activity, but RpoS remains inactive, limiting *vpvABC* activation to VpsT. Conversely, in the interior, more similar to the stationary phase, RpoS becomes active, and together with VpsT, promotes *vpvABC* transcription despite potentially lower c-di-GMP levels. Another explanation for this observation could be due to impaired GFP folding due to limited oxygen availability in thicker parts of the biofilms. While we have not conducted a formal analysis of oxygen gradients or their impact on GFP fluorescence, previous studies have shown that in biofilms thicker than 50 μm, no lateral or vertical fluorescence gradients were detectable, and GFP fluorescence developed uniformly throughout the biofilm [60,61]. All biofilms analyzed in this study were within this range of thickness. Thus, we link the differences in fluorescence ratios to genetic variation rather than to differences in biofilm thickness.

In *V. cholerae*, we are beginning to better understand the transcriptional regulation of DGCs and PDEs. The major biofilm regulators VpsR and VpsT control the expression of diverse DGCs and PDEs, with several having direct binding sites in their promoter region – such as VpsT’s direct regulation of *vpvC* [42,53,55]. Beyond these c-di-GMP-responsive transcription factors, additional regulators directly or indirectly control expression of c-di-GMP metabolism genes. This includes the major transcriptional repressor of biofilm formation HapR, the catabolite repressor protein CRP, and the histone-like nucleoid structuring protein H-NS [53,62–65]. This observation suggests that some DGCs and PDEs are regulated by multiple transcriptional regulators activating biofilm formation, illustrating that they are part of the core c-di-GMP network. Our findings expand this regulatory network by demonstrating RpoS-mediated regulation of an additional 35 genes encoding DGCs and PDEs. While most of these genes are down regulated in the absence of RpoS, two genes VCA0101, which encode an EAL domain protein, and VCA0165, a GGDEF domain protein, are upregulated. However, their biological functions remain largely unknown. Of this c-di-GMP RpoS regulon, we found that RpoS directly binds to the promoter of *vpvABC* and 18 other c-di-GMP-related genes. These include seven predicted GGDEF-domain diguanylate cyclases (VC1029 - *cdgB*, VC1370, VC2285 - *cdgL*, VC2454 - *vpvC*, VCA0557, VCA0560, VCA0965), four EAL-domain phosphodiesterases (VC1086, VC1641, VC1710 - *pdeS*, VC1851), three HD-GYP-domain phosphodiesterases (VC1087, VC2340, VCA0210), and five hybrid GGDEF–EAL domain proteins (VC0658 - *cdgI*, VC1934, VC2750, VCA0080, VCA1082-83 - *lapD*). Several of these have known roles in controlling biofilm formation and motility [8]. The DGCs VpvC, CdgB, CdgL, CdgN, VCA0560, and VCA0965 promote matrix production while repressing flagellar motility. PDEs such as VC1086, VC1710, and VCA0210 reduce c-di-GMP levels and promote motility/ dispersal.

We found that only VpvC and CdgA contribute to colony rugosity phenotype under the conditions tested. Previous work has shown that overexpression of c-di-GMP–producing proteins alone is not sufficient to induce rugosity. Rather, several factors seem to be required for this phenotype, including the *vpvC*^W240R^ mutation, loss of HapR and the resulting de-repression of *cdgA*, and disruption of flagellar function [35,38,53]. In this context, deletion of *rpoS* reduces rugosity in our mutant strain, likely due to decreased transcription of *vpvC*^W240R^. We also find that overexpression of *cdgA* can promote rugosity and that RpoS contributes to *cdgA* expression. However, HapR continues to repress *cdgA* when it is present, and RpoS positively regulates *hapR* transcription during stationary phase. Therefore, in our rugose strain, the contribution of RpoS to rugosity appears to occur primarily through its regulation of *vpvC*. Although the mechanism by which the *vpvC*^W240R^ mutation enhances diguanylate cyclase activity remains unclear, these findings suggest that RpoS may affect multiple pathways associated with rugosity through transcriptional control.

In *V. cholerae*, RpoS positively regulates motility and biofilm dispersal, suggesting that RpoS acts in opposition to biofilm formation [46,56,59]. However, our findings show that RpoS can also positively regulate transcription of DGCs and PDEs, suggesting that RpoS plays a more complex role in *V. cholerae* c-di-GMP signaling than previously thought. The RpoS regulon has many genes encoding c-di-GMP metabolizing enzymes whose roles in regulating cellular levels of c-di-GMP and biofilm formation have yet to be determined [40]. Some DGCs and PDEs are directly regulated by RpoS, which are likely expressed in response to stress or environmental conditions. Some of these include the SOS-induced VC1370 [66], iron- and quorum sensing-regulated VCA0560 [67], and sugar-responsive VC1710 (PdeS) [68]. RpoS also targets the promoter of LapD (VCA1082–1083), which does not produce or degrade c-di-GMP but instead binds it to modulate FrhA and CraA adhesin levels at the surface and influence biofilm formation [24]. Taken together, the direct regulation of a wide range of DGCs and PDEs, along with c-di-GMP receptors like LapD - supports a role for RpoS in fine-tuning cellular c-di-GMP pools, and thus biofilm formation and motility, in response to environmental stresses and growth phase.

*E. coli* also expresses DGCs and PDEs in a RpoS-dependent manner [47,69]. Genome-wide transcriptional investigations show that RpoS controls about 10% of *E. coli* genes directly or indirectly under various stress conditions. Approximately 10% of them encode regulatory or signal transduction proteins. Notably, the expression of seven of the 29 GGDEF/EAL domain-containing genes is upregulated by RpoS, whereas none are significantly downregulated [69]. These findings highlight the role of c-di-GMP signaling in stress adaptation and the post-exponential growth phase. The more we understand the intersection of c-di-GMP specificity with environmental challenges, the better we can conceptualize and target specific enzymes linked to strategies that promote survival and transmission of human pathogens.

## Materials and methods

### Bacterial strains and growth conditions

The strains and plasmids used in this study are listed in S1 Table. *E. coli* DH5α-λpir strains were used for DNA manipulation, and *E. coli* S17-1 λpir strains were used for conjugation with *V. cholerae. V. cholerae* and *E. coli* strains were grown aerobically in Luria-Bertani Miller (LB) broth (1% tryptone, 0.5% yeast extract, 1% NaCl [pH 7.5]) at 30˚C and 37˚C, respectively. LB agar medium included granulated agar (BD Difco, Franklin Lakes, NJ) at 1.5% (wt/vol). Antibiotics were used at the following concentrations when applicable: ampicillin (Ap), 100 μg/mL; rifampicin (Rif), 100 μg/mL; gentamicin (Gm), 15 μg/mL; kanamycin (Kan), 50 µg/mL; chloramphenicol (Cm), 5 μg/mL or 2.5 μg/mL for *V. cholerae* during luminescence assays. For overnight cultures, strains were struck from frozen glycerol stock onto an LB-agar plate and grown at 30˚C overnight, 5 colonies were then inoculated into 5 mL LB media and grown overnight at 30˚C with aeration (200 rpm).

### Strain and plasmid construction

Plasmids were constructed using standard molecular cloning techniques or the Gibson Assembly recombinant DNA technique (New England BioLabs, Ipswich, MA). DNA sequences were confirmed using Genewiz from Azenta Life Sciences. In-frame gene deletions were generated through allelic exchange of native open reading frame (ORF) with the truncated ORF, as previously described [70].

### Luminescence and c-di-GMP biosensor assay

*V. cholerae* strains harboring transcriptional reporter P*vpvA-luxCDABE* were grown overnight with aeration in LB broth supplemented with chloramphenicol 5 μg/mL. Cultures were diluted 1:500 into 10 mL of fresh LB in flasks containing 2.5 μg/mL chloramphenicol. The freshly inoculated cultures were grown aerobically at 30˚C to exponential growth phase (OD600 of 0.3) and stationary growth phase (OD600 of 4.0), then luminescence was measured. For Fig 1B, A1552 Ptac-VCA0956 was induced with the appropriate IPTG concentration following 1:500 dilution. For the c-di-GMP biosensor, *V. cholerae* strains harboring pFY4535 were diluted 1:500 into 10 mL of fresh LB in flasks. The freshly inoculated cultures were grown aerobically at 30˚C to stationary growth phase (OD600 of 4.0) then TurboRFP and AmCyan fluorescent intensities were measured. Fluorescence and luminescence measurements were performed using a PerkinElmer EnVision 2105 multimode plate reader (PerkinElmer, Waltham, MA).

### Transposon mutagenesis screen

Transposon libraries were created in the rugose background as previously described [71]. P*vpvA-sfGFP* was mated into the R::mTn10 library, serially diluted, and plated onto 1.5% agar LB 50 µg/mL kanamycin, 100 μg/mL rifampicin, and 15 μg/mL gentamicin. Rugose variant with P*vpvA-sfGFP* were serially diluted and plated on 1.5% agar LB containing 100 μg/mL rifampicin and 15 μg/mL gentamicin to determine which colonies with mTn10 insertions had unaltered *vpvABC* transcription. Plates were incubated at 30°C for 48 hours and checked for changes in fluorescence. Candidate mutants were streak purified and compared against the rugose parental strain containing P*vpvA-sfGFP* as well as the empty vector (promoterless-sfGFP). Transposon insertion sites were identified via arbitrary PCR and Sanger sequencing (Genewiz from Azenta Life Sciences) as previously described [51].

### c-di-GMP quantification

*V. cholerae* strains were grown overnight with aeration in LB broth. Cultures were diluted 1:200 into 2x50 mL of fresh LB in flasks and then grown aerobically at 30°C. Growth was monitored and cultures were harvested at the stationary growth phase (OD600 of 4.0). Cultures were harvested for nucleotide extraction (10 mL) and total protein abundance via BCA assay (1 mL). For nucleotide extraction, cell pellets were resuspended in 1 mL of extraction buffer (40% acetonitrile, 40% methanol, 0.1% formic acid, 19.9% HPLC grade H2O) and vortexed for 30 seconds. Insoluble components were spun down at 16,000 g for 5 minutes, and 800 μL of the supernatant was collected and dried under vacuum. The dried sample was then resuspended in 50 μL HPLC grade H2O containing 184 mM NaCl, and c-di-GMP was quantified via LC-MS/MS at the UCSC Chemistry and Biochemistry Mass Spectrometry facility [36]. c-di-GMP standard curves were generated using c-di-GMP standards (SIGMA) of 25, 50, 100, 500, 2000, 3500, and 5000 nM dissolved in HPLC grade H2O containing 184 mM NaCl. The abundance of c-di-GMP was extrapolated from the mass spectroscopy data and normalized to protein abundance.

### Analysis of colony corrugation

20 mL of 1.5% agar LB were poured per plate and allowed to dry 2 days before use. Strains were grown overnight with aeration in LB broth. Cultures were diluted 1:200 in LB and 2 μL was spotted in triplicate onto 1.5% agar LB plates. Plates were incubated for 48 or 72 hours at 30°C and imaged using a Zeiss Stemi 2000-C microscope equipped with Zeiss AxioCam ERc 5 s Microscope Camera. A minimum of two biological replicates were performed per experiment.

### Live biofilm imaging

Inoculation of flow cells was done by diluting overnight-grown cultures 1:200 in 2% v/v LB (0.02% tryptone, 0.01% yeast extract, 1% NaCl; pH 7.5) and injecting into a μ-Slide VI 0.4 uncoated (Ibidi, Martinsried, Germany). Following injection, attachment to the flow cell surface was allowed for 1 hour at 30°C. Flow of 2% v/v LB was initiated at a rate of 7.5 mL/hour and continued for 24 hours. Live biofilms were imaged with a 40x dry numerical aperture 0.8 objective using a Zeiss 880 Confocal microscope with Airyscan Fast with an image size of 1128 x 1128 pixels (141.70 µm x 141.70 µm, scaled). For mCardinal P*vpvA-sfGFP*, gentamicin was supplemented into 2% v/v LB, a 488-nm laser was used to excite sfGFP, and 594-nm laser for mCardinal. For pFY4950, a 561-nm laser was used to excite TurboRFP and a 458-nm laser for AmCyan. At least three Z-stacks were taken at independent locations within at least two biofilm replicates for image analysis.

### Image analysis

Quantitative image analysis was performed using BiofilmQ, a software tool that has been developed for measuring spatially-resolved biofilm properties (available at drescherlab.org/data/BiofilmQ), details can be found in the cited publication and website [72]. Images were segmented with a threshold value of 0.15. Following segmentation, the parameters calculated were global biofilm properties, surface properties, and intensity properties. For spatial quantification of *vpvA* expression (sfGFP/mCardinal) or c-di-GMP abundance (TurboRFP/amCyan), fluorescent mean intensity ratios were calculated as previously described [51]. Kymographs of fluorescent mean intensity ratios as a function of biofilm depth were obtained by averaging over the biological and technical replicates (n = 6).

### RNA Isolation

*V. cholerae* strains were grown overnight to an OD_600_ of 4.0 in LB broth at 30˚C and then diluted 1:200 into fresh LB medium. The diluted sample was spotted onto LB agar plates and incubated for 48 hours at 30˚C to grow colony biofilms, which were then harvested using sterile 10 µL inoculation loops and immediately resuspended in 1 mL TRIzol (Invitrogen). The samples were vortexed to disperse the colony biofilm, then flash-frozen, and stored at -80˚C until RNA isolation. Total RNA isolation was performed according to the TRIzol manufacturer’s instructions. 500 µL of high salt buffer (0.8 M sodium citrate and 1.2 M sodium chloride) was added to 500 µL isopropanol post-aqueous separation to handle matrix contamination from the biofilm. DNase treatment, rRNA depletion (RiboZero Plus, Illumina), and library prep (Stranded RNA library preparation, Illumina) were performed according to the manufacturer’s instructions. Sample QC was performed via Bioanalyzer. Illumina sequencing was performed for paired-end 150 bp reads.

### RNA-sequencing data analysis

Quality checks were performed on read data with FASTQC, version 0.11.9 [73]. Trimming was not performed as deemed unneeded from the FASTQC analysis output. Transcript abundance was quantified with Salmon [74], version 1.5.2, using a recently inferred *V. cholerae* transcriptome derived from the N16961 reference genome (NC_002505.1 and NC_002506.1) as an index [66]. The resulting salmon quantification files were imported into R via tximeta, version 1.22.1 [75]. Read count normalization and differential expression analysis were performed using DESeq2, version 1.32.0 [76]. Gene set enrichment analysis was performed using fgsea, version 1.30.0 [77].

### ChIP-seq library preparation and sequencing

These experiments were done as previously described [78]. Briefly, the *V. cholerae* A1552 Δ*rpoS* chromosome was altered to encode *rpoS* with a C-terminal 3xFLAG fusion. This strain, and the parent, were each grown aerobically to OD_600_ of 2.0 in LB broth at 37^°^C and crosslinked using formaldehyde. Nucleoprotein was then extracted and sheared exactly as in our prior work [78]. After immunoprecipitation using anti-FLAG antibodies, protein-DNA complexes were washed to remove non-specific interactions before preparing libraries for Illumina sequencing. The raw data are assigned to the accession code E-MTAB-14434 and are available from ArrayExpress.

### ChIP-seq bioinformatics

Sequencing reads were aligned to *V. cholerae* N16961 genome (Genbank accession numbers CP024162.1 and CP024163.1) using Bowtie 2 [79]. The resulting BAM files were processed using the multiBamSummary (Galaxy Version 3.5.4) function of deepTools2 to calculate read coverage per base across the genome [80]. To identify RpoS bound regions we first normalized all data, so the average read depth was 1. We then determined the average read depth per base between replicates. The read depth for the control strain was then subtracted from that generated using the strain encoding RpoS-3xFLAG. To select peaks, we identified regions where final signal was 1.25 or more, over a region of at least 200 bp, using Artemis [81]. Annotation of peaks with their nearest RefSeq gene promoter was performed using annotatePeaks.pl from HOMER, version 4.11 [82]. To create circular plots of ChIP-seq data for each chromosome we used DNAPlotter [83].

### Operon structure phylogenetic analysis

The conservation of *vpvABC* genes was evaluated using the operon_conserve_detect pipeline (https://github.com/ErillLab/oprn_consv_calc), as reported previously [84]. The pipeline automates tBLASTN searches of each provided protein from a reference locus against the NCBI RefSeq representative genomes database (ref_prok_rep_genomes). Searches were limited to the taxonomic group Pseudomonadota (txid:1224), with hits filtered to a maximum e-value of 1e − 10 and a minimum coverage threshold of 30%. To confirm homology, each hit was further validated through reverse BLASTP against the original reference genome. The resulting nucleotide records were grouped by genome using their assembly accession numbers. For each record, hits were grouped into operons when satisfying three criteria: (1) all genes must lie on the same DNA strand, (2) a maximum of three annotated features may separate any two hits, and (3) the intergenic distance between adjacent genes must be under 150 base pairs. Locus structure conservation was quantified by calculating the split distance of each locus relative to the reference [84], yielding a value between 0 (no conservation) and 1 (full conservation). Structural similarity scores and percent identity values for each gene were annotated onto a reference phylogenetic tree using the iTOL web tool [85]. This tree was built using homologs of *Escherichia coli* RecA (WP_000963143.1), identified via BLASTP across the species of interest using the phylo_seq_gen Python script (https://github.com/ErillLab/phylo_seq_gen). The RecA sequences were aligned using CLUSTALW, and used to construct a maximum likelihood tree (100 bootstrap replicates) using the build function of ETE3 3.1.3 [86] as implemented on the GenomeNet server (https://www.genome.jp/tools/ete/). A maximum likelihood tree was inferred using RAxML v8.2.11 ran with model PROTGAMMAJTT and default parameters [87]. The reference phylogeny was annotated using the iTOL web suite [88].

## Supporting information

S1 Table Strains and plasmids used in this study. (DOCX)

S2 Table Transposon mutagenesis screen of *vpv* regulators. List of transposon mutants with altered fluorescence indicating gene mutated, number of unique insertions, and impact on fluorescence from pFY7140. (-) indicates a decrease in fluorescence compared to the pNUT542-P*vpvA*-*sfGFP* control and (+) indicates an increase. The number of symbols corresponds to the degree of relative fluorescent change.
(DOCX)

S1 Fig Altered *vpvA* promoter activity in rugose mTn10 mutants via P*vpvA*-*sfGFP.* Fluorescence intensity of sfGFP under the control of the *vpvA* promoter with cells grown for 48 hours on solid growth media agar plates at 30 °C. Rugose with P*vpvA*-sfGFP and empty reporters were used to adjust image quality as a middle point and low point for the heat spectrum respectively. Insertions in *vpvA*, *vpsT*, *vpsR*, *rpoS*, and *rssB* are shown from isolated mutants identified in the transposon mutagenesis screen.
(TIF)

S2 Fig RpoS controls a large portion of the *V. cholerae* transcriptome. (A) Volcano plot of differentially regulated genes in RΔ*rpoS* compared to rugose showing relative fold change (log2FC) for each gene plotted against the associated adjusted p-value (-log10(p.adj)). Genes are color coded based on their association to various cellular systems: biofilm formation (green), c-di-GMP metabolism (orange), chemotaxis (purple), flagellar assembly (dark grey), nucleotide metabolism (teal), oxidative stress (pink), pathogenesis (yellow), and T6SS (brown). Colored vertical lines along the x-axis (rug plot) indicate the distribution of genes from each functional pathway, matching the colors of corresponding points in the volcano plot. (B) Volcano plots for each of these systems with their associated color coding including a plot of all other genes (light grey). (C) Gene set enrichment analysis (GSEA) using GO biological process pathways was performed using differentially regulated genes in R∆*rpoS* compared to rugose. The top enriched GO groups and their normalized enrichment scores are shown. The size of the point represents the number of genes in a particular pathway that were differentially regulated, while the color denotes the adjusted p-value.
(TIF)

S3 Fig Impact of RpoS regulated DGCs on rugosity. Representative images of deletion mutants of RpoS regulated DGCs in the rugose background. Colonies were grown for 72 hours at 30°C.
(TIF)

S4 Fig RpoS regulates DGCs and PDEs in a wild-type background. Volcano plot of differentially regulated c-di-GMP metabolism genes in Δ*rpoS* compared to the wild-type. Genes with GGDEF domains are represented by green upward triangles, GGDEF-EAL domains by orange diamonds, EAL domains by red downward triangles, and HD-GYP domains by red empty downward triangles. Dotted lines indicate a log2FC threshold of -1 and 1.
(TIF)

S5 Fig Conservation of the *vpvABC* operon. Conservation of the *vpvABC* operon across the Pseudomonadota, annotated in the context of a RecA phylogeny. All *Vibrio* reference genomes and all genomes with an average percent identity above 15% for *vpv* operon genes are represented. The percent amino acid conservation for the homologs of each Vpv operon component and the structural similarity for the entire operon (i.e., gene synteny) are presented as colored gradients.
(PDF)
