# Supplementary material for: Parallel regulatory circuits orchestrate biofilm formation in response to c-di-GMP levels and growth phase
Source: PLoS Genet. 2025 Sep 15;21(9):e1011870. doi: 10.1371/journal.pgen.1011870 (PMC12456836; doi:10.1371/journal.pgen.1011870)
Supplement: S1 Table — (DOCX) [file pgen.1011870.s001.docx]

| **S1 Table. Strains and plasmids used in this study** | | | |
| --- | --- | --- | --- |
| FY# | *V. cholerae* strains | Resistance | Reference |
| FY_VC_1 | O1 El Tor A1552 | Rif | (1) |
| FY_VC_2 | O1 El Tor A1552, rugose variant | Rif | (1) |
| FY_VC_1745 | FY_VC_2 Δ*vpvC* | Rif | (2) |
| FY_VC_5 | FY_VC_2 Δ*vpsT*Δ*lacZ* | Rif | (3) |
| FY_VC_6 | FY_VC_2 Δ*vpsR*Δ*lacZ* | Rif | (3) |
| FY_VC_16716 | FY_VC_2 Δ*rpoS* | Rif | This study |
| FY_VC_17190 | FY_VC_2 Δ*rpoS lacZ::rpoS* | Rif | This study |
| FY_VC_18213 | FY_VC_2 Δ*rssB* | Rif | This study |
| FY_VC_17193 | FY_VC_5 Δ*rpoS* | Rif | This study |
| FY_VC_17194 | FY_VC_6 Δ*rpoS* | Rif | This study |
| FY_VC_18215 | FY_VC_2 Δ*vpsT*Δ*rssB* | Rif | This study |
| FY_VC_9917 | FY_VC_1 VCA0956::P*lacIq-lacI* Ptac-VCA0956 | Rif | (4) |
| FY_VC_17198 | FY_VC_2, pFY7138 | Rif, Cm | This study |
| FY_VC_17199 | FY_VC_1745, pFY7138 | Rif, Cm | This study |
| FY_VC_17200 | FY_VC_5, pFY7138 | Rif, Cm | This study |
| FY_VC_17201 | FY_VC_6, pFY7138 | Rif, Cm | This study |
| FY_VC_17202 | FY_VC_16716, pFY7138 | Rif, Cm | This study |
| FY_VC_17227 | FY_VC_17190, pFY7138 | Rif, Cm | This study |
| FY_VC_18218 | FY_VC_18213, pFY7138 | Rif, Cm | This study |
| FY_VC_17204 | FY_VC_17193, pFY7138 | Rif, Cm | This study |
| FY_VC_17226 | FY_VC_17194, pFY7138 | Rif, Cm | This study |
| FY_VC_18219 | FY_VC_18215, pFY7138 | Rif, Cm | This study |
| FY_VC_17230 | FY_VC_9917, pFY7138 | Rif, Cm | This study |
| FY_VC_17231 | FY_VC_2, pFY7106 | Rif, Gm | This study |
| FY_VC_17232 | FY_VC_1745, pFY7106 | Rif, Gm | This study |
| FY_VC_17233 | FY_VC_5, pFY7106 | Rif, Gm | This study |
| FY_VC_17234 | FY_VC_6, pFY7106 | Rif, Gm | This study |
| FY_VC_17235 | FY_VC_16716, pFY7106 | Rif, Gm | This study |
| FY_VC_17236 | FY_VC_17190, pFY7106 | Rif, Gm | This study |
| FY_VC_17878 | FY_VC_17193, pFY7106 | Rif, Gm | This study |
| FY_VC_17880 | FY_VC_17194, pFY7106 | Rif, Gm | This study |
| FY_VC_17905 | FY_VC_2, pFY4950 | Rif, Gm | This study |
| FY_VC_17906 | FY_VC_1745, pFY4950 | Rif, Gm | This study |
| FY_VC_17907 | FY_VC_5, pFY4950 | Rif, Gm | This study |
| FY_VC_17908 | FY_VC_6, pFY4950 | Rif, Gm | This study |
| FY_VC_17909 | FY_VC_16716, pFY4950 | Rif, Gm | This study |
| FY_VC_17910 | FY_VC_17190, pFY4950 | Rif, Gm | This study |
| FY_VC_17879 | FY_VC_17193, pFY4950 | Rif, Gm | This study |
| FY_VC_17881 | FY_VC_17194, pFY4950 | Rif, Gm | This study |
| FY_VC_8902 | FY_VC_2, pFY1054 | Rif, Amp | (5) |
| FY_VC_18220 | FY_VC_16716, pFY1054 | Rif, Amp | This study |
| FY_VC_18272 | FY_VC_16716, pFY7245 | Rif, Amp | This study |
| FY_VC_18221 | FY_VC_16716, pFY7210 | Rif, Amp | This study |
| FY_VC_18222 | FY_VC_16716, pFY4350 | Rif, Amp | This study |
| FY_VC_18271 | FY_VC_16716, pFY7252 | Rif, Amp | This study |
| FY_VC_18223 | FY_VC_16716, pFY7211 | Rif, Amp | This study |
| FY_VC_18303 | FY_VC_1 RpoS-3xFLAG | Rif, Amp | This study |
| Plasmids | | | |
| pDL1093 | Temperature sensitive mTn10 delivery vector | Kan, Cm | (6) |
| pGP704sacB28 | pGP704 derivative, *mob*/*oriT* *sacB*, Ampr | Amp | G. Schoolnik |
| pFY314 | pGP704sacB28::Δ*rpoS* | Amp | (7) |
| pFY7141 | pGP704sacB28 *lacZ::rpoS* | Amp | This study |
| pFY1839 | pGP704sacB28::*ΔrssB* | Amp | (8) |
| pFY957 | pBBR MCS-*luxCDABE* | Cm | (9) |
| pFY7138 | pBBR Promoter(*vpvA*)-*luxCDABE* | Cm | This study |
| pNUT542 | pNUT backbone, Ptac-*sfGFP* | Gm | (10) |
| pFY7143 | pNUT542 promoterless-*sfGFP* | Gm | This study |
| pFY7140 | pNUT542 Promoter(*vpvA*)-*sfGFP* | Gm | This study |
| pFY7104 | pNUT backbone, constitutively expressed *mCardinal*, multicloning site upstream of *sfGFP* tagged with AAV degron | Gm | This study |
| pNUT883 | pNUT sfGFP MCS-*mRuby2* | Gm | (10) |
| pFY7106 | pFY7104 *mCardinal* and Promoter(*vpvA*)-*sfGFP*-AAV | Gm | This study |
| pFY4535 | pMMB Gm(r) *amcyan*-Bc34-*turboRFP* (*hok-sok*) | Gm | (11) |
| pFY4950 | pMMB Gm(r) *amcyan*-Bc34-*turboRFP*-AAV (*hok-sok*) | Gm | This study |
| pFY1054 | pMMB67EH | Amp | (12) |
| pFY7245 | pMMB67EH -*vpvC* | Amp | This study |
| pFY7210 | pMMB67EH -*vpvC*(W240R) | Amp | This study |
| pFY4350 | pMMB67EH-VCA0956 | Amp | This study |
| pFY7211 | pMMB67EH-VC1029 | Amp | This study |
| pFY7252 | pMMB67EH -VCA0074 (*cdgA*) | Amp | This study |
| pFY7236 | pGP704 for RpoS 3xFLAG knock in | Amp | This study |

1. Yildiz FH, Schoolnik GK. *Vibrio cholerae* O1 El Tor: identification of a gene cluster required for the rugose colony type, exopolysaccharide production, chlorine resistance, and biofilm formation. Proc Natl Acad. Sci. USA. 1999 Mar 30;96(7):4028-33.
2. Beyhan S, Odell LS, Yildiz FH. Identification and Characterization of Cyclic Diguanylate Signaling Systems Controlling Rugosity in *Vibrio cholerae*. J Bacteriol. 2008 Nov;190(22):7392-405.
3. Casper-Lindley C, Yildiz FH. VpsT Is a Transcriptional Regulator Required for Expression of *vps* Biosynthesis Genes and the Development of Rugose Colonial Morphology in *Vibrio cholerae* O1 El Tor. J Bacteriol. 2004 Mar;186(5):1574-8.
4. Jones CJ, Utada A, Davis KR, Thongsomboon W, Sanchez DZ, Banakar V, Cegelski L, Wong G.C.L, Yildiz F.H. C-di-GMP Regulates Motile to Sessile Transition by Modulating MshA Pili Biogenesis and Near-Surface Motility Behavior in *Vibrio cholerae*. PLoS Pathog. 2015 Oct 27;11(10):e1005068.
5. Giglio KM, Fong JC, Yildiz FH, Sondermann H. Structural Basis for Biofilm Formation via the *Vibrio cholerae* Matrix Protein RbmA. J Bacteriol. 2013 Jul;195(14):3277_86.
6. Duncan MC, Forbes JC, Nguyen Y, Shull LM, Gillette RK, Lazinski DW, Ali A, Shanks RMQ, Kadouri DE, Camilli A. *Vibrio cholerae* motility exerts drag force to impede attack by the bacterial predator *Bdellovibrio bacteriovorus*. Nat Commun. 2018 Nov 12;9(1):4757.
7. Shikuma NJ, Yildiz FH. Identification and Characterization of OscR, a Transcriptional Regulator Involved in Osmolarity Adaptation in *Vibrio cholerae*. J Bacteriol. 2009 Jul;191(13):4082–96.
8. Cheng AT, Ottemann KM, Yildiz FH. *Vibrio cholerae* Response Regulator VxrB Controls Colonization and Regulates the Type VI Secretion System. PLoS Pathog. 2015 May 22;11(5):e1004933.
9. Lenz DH, Mok KC, Lilley BN, Kulkarni RV, Wingreen NS, Bassler BL. The Small RNA Chaperone Hfq and Multiple Small RNAs Control Quorum Sensing in *Vibrio harveyi* and *Vibrio cholerae*. Cell. 2004 Jul 9;118(1):69-82.
10. Singh PK, Bartalomej S, Hartmann R, Jeckel H, Vidakovic L, Nadell CD, Drescher K. *Vibrio cholerae* Combines Individual and Collective Sensing to Trigger Biofilm Dispersal. Curr Bio. 2017 Nov 6;27(21):3359-3366.e7.
11. Zamorano-Sánchez D, Xian W, Lee CK, Salinas M, Thongsomboon W, Cegelski L, Wong GCL, Yildiz FH. Functional Specialization in *Vibrio cholerae* Diguanylate Cyclases: Distinct Modes of Motility Suppression and c-di-GMP Production. mBio. 2019 Apr 23;10(2):e00670-19.
12. Fürste JP, Pansegrau W, Frank R, Blöcker H, Scholz P, Bagdasarian M, Lanka E. Molecular cloning of the plasmid RP4 primase region in a multi-host-range *tacP* expression vector. Gene. 1986 Jan 1;48(1):119-31.
