# Supplementary material for: Parallel regulatory circuits orchestrate biofilm formation in response to c-di-GMP levels and growth phase
Source: PLoS Genet. 2025 Sep 15;21(9):e1011870. doi: 10.1371/journal.pgen.1011870 (PMC12456836; doi:10.1371/journal.pgen.1011870)
Supplement: S2 Table — List of transposon mutants with altered fluorescence indicating gene mutated, number of unique insertions, and impact on fluorescence from pFY7140. (-) indicates a decrease in fluorescence compared to the pNUT542-PvpvA-sfGFP control and (+) indicates an increase. The number of symbols corresponds to the degree of relative fluorescent change. (DOCX) [file pgen.1011870.s002.docx]

| **S2 Table. Transposon mutagenesis screen of *vpv* regulators.** | | |
| --- | --- | --- |
| **Gene** | **Number of Insertions** | **Impact on *vpv* expression** |
| VC0250 | 1 | ++ |
| VC0251 | 1 | + |
| VC0269 | 1 | ++ |
| **VC0534 (*rpoS*)** | **4** | **---** |
| VC0665 (*vpsR*) | 1 | -- |
| VC0916 (*vpsU*) | 1 | -- |
| VC1050 (*rssB*) | 1 | +++ |
| VC1142 (*cspD*) | 2 | - |
| VC1601 | 1 | - |
| VC1764 | 1 | -- |
| VC1770 (*ddmE*) | 2 | +++ |
| VC1771 (*ddmD*) | 7 | +++ |
| VC1796 | 1 | + |
| VC2456 (*vpvA*) | 3 | --- |
| VCA0011 (*malT*) | 1 | ++ |
| VCA0729 | 1 | -- |
| VCA0952 (*vpsT*) | 3 | -- |
