# Supplementary figures and images for: Parallel regulatory circuits orchestrate biofilm formation in response to c-di-GMP levels and growth phase
Source: PLoS Genet. 2025 Sep 15;21(9):e1011870. doi: 10.1371/journal.pgen.1011870 (PMC12456836; doi:10.1371/journal.pgen.1011870)

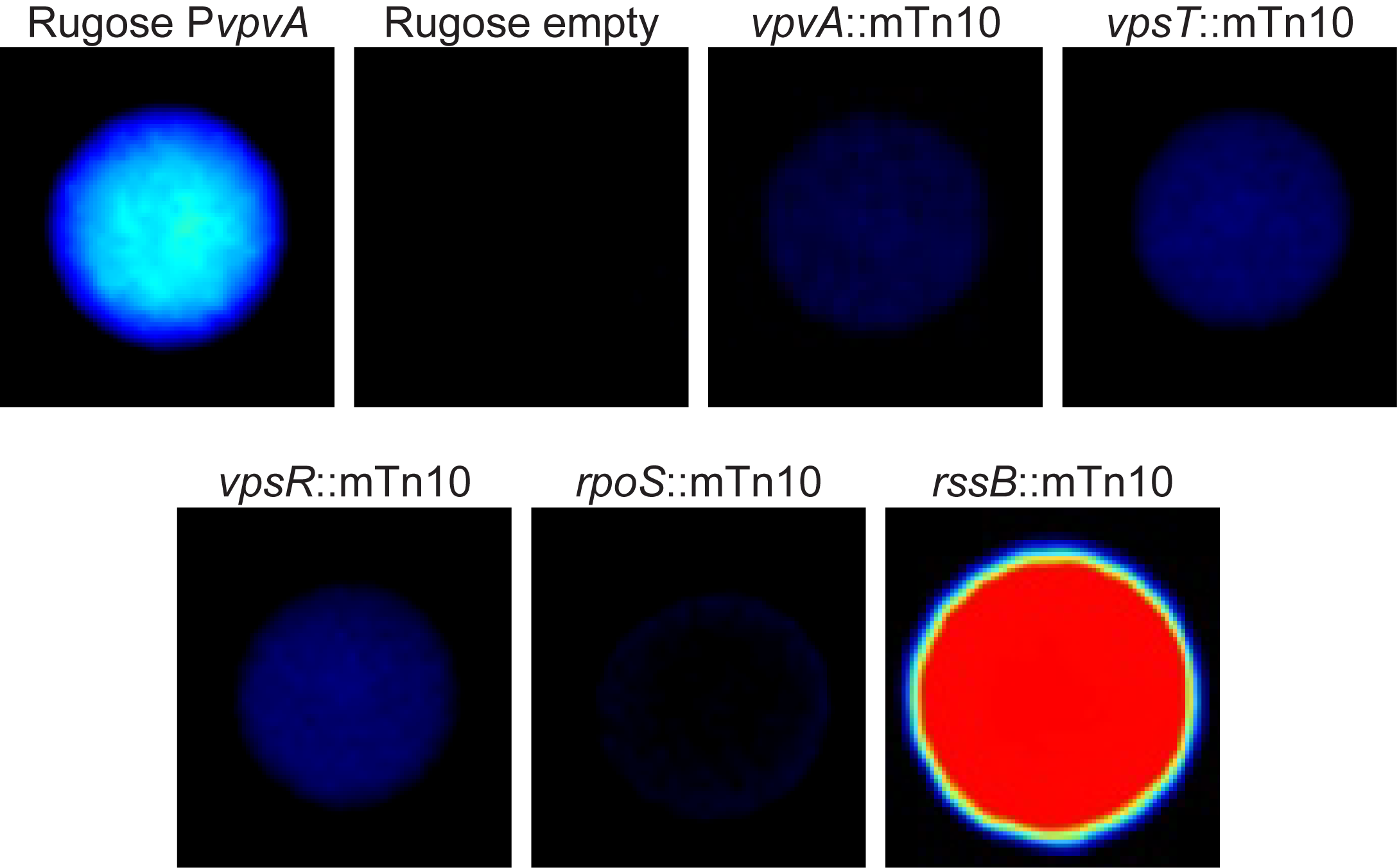

Supplement: S1 Fig — Fluorescence intensity of sfGFP under the control of the vpvA promoter with cells grown for 48 hours on solid growth media agar plates at 30 °C. Rugose with PvpvA-sfGFP and empty reporters were used to adjust image quality as a middle point and low point for the heat spectrum respectively. Insertions in vpvA, vpsT, vpsR, rpoS, and rssB are shown from isolated mutants identified in the transposon mutagenesis screen. (TIF) [file pgen.1011870.s003.tif]

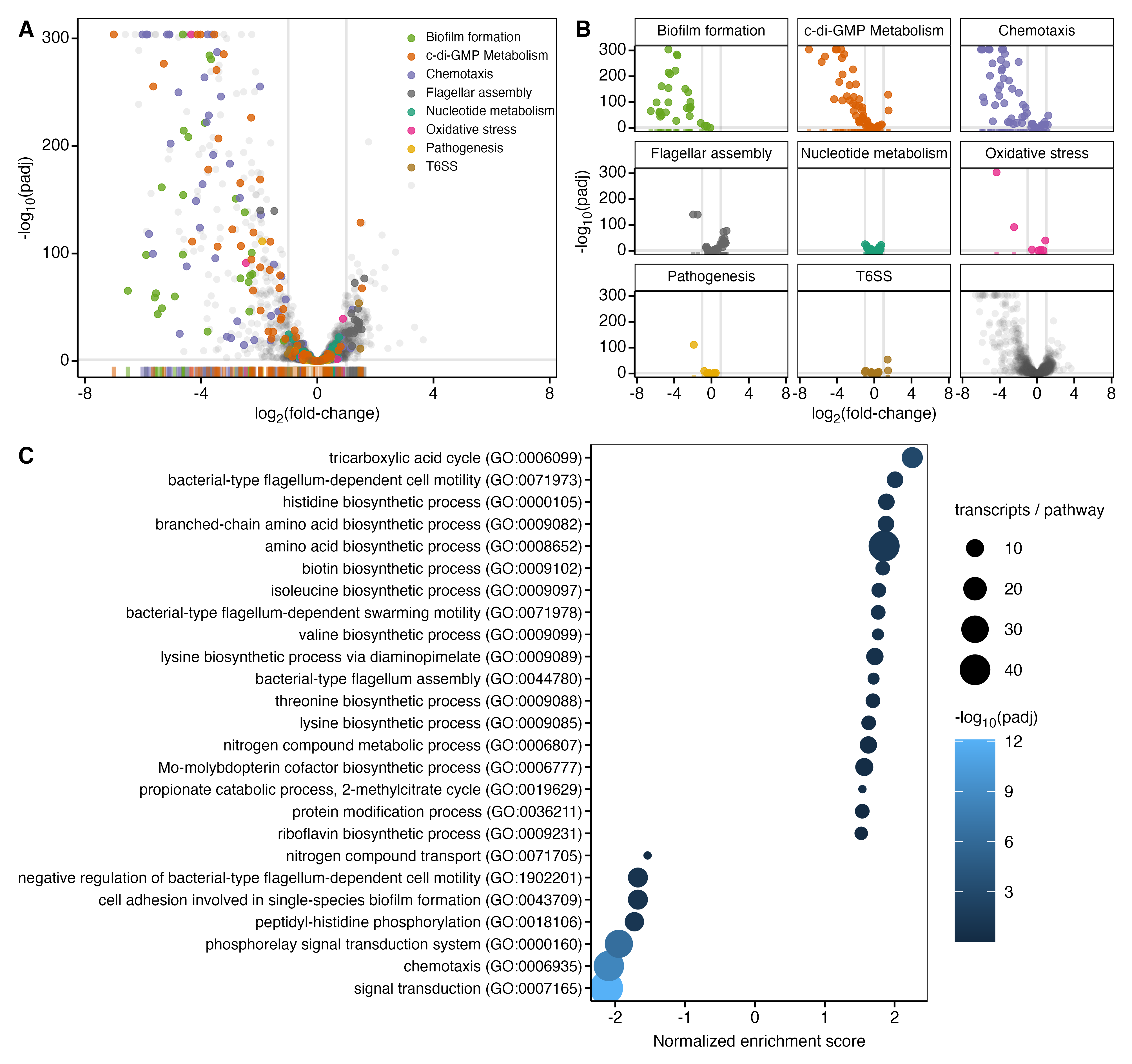

Supplement: S2 Fig — (A) Volcano plot of differentially regulated genes in RΔrpoS compared to rugose showing relative fold change (log2FC) for each gene plotted against the associated adjusted p-value (-log10(p.adj)). Genes are color coded based on their association to various cellular systems: biofilm formation (green), c-di-GMP metabolism (orange), chemotaxis (purple), flagellar assembly (dark grey), nucleotide metabolism (teal), oxidative stress (pink), pathogenesis (yellow), and T6SS (brown). Colored vertical lines along the x-axis (rug plot) indicate the distribution of genes from each functional pathway, matching the colors of corresponding points in the volcano plot. (B) Volcano plots for each of these systems with their associated color coding including a plot of all other genes (light grey). (C) Gene set enrichment analysis (GSEA) using GO biological process pathways was performed using differentially regulated genes in R∆rpoS compared to rugose. The top enriched GO groups and their normalized enrichment scores are shown. The size of the point represents the number of genes in a particular pathway that were differentially regulated, while the color denotes the adjusted p-value. (TIF) [file pgen.1011870.s004.tif]

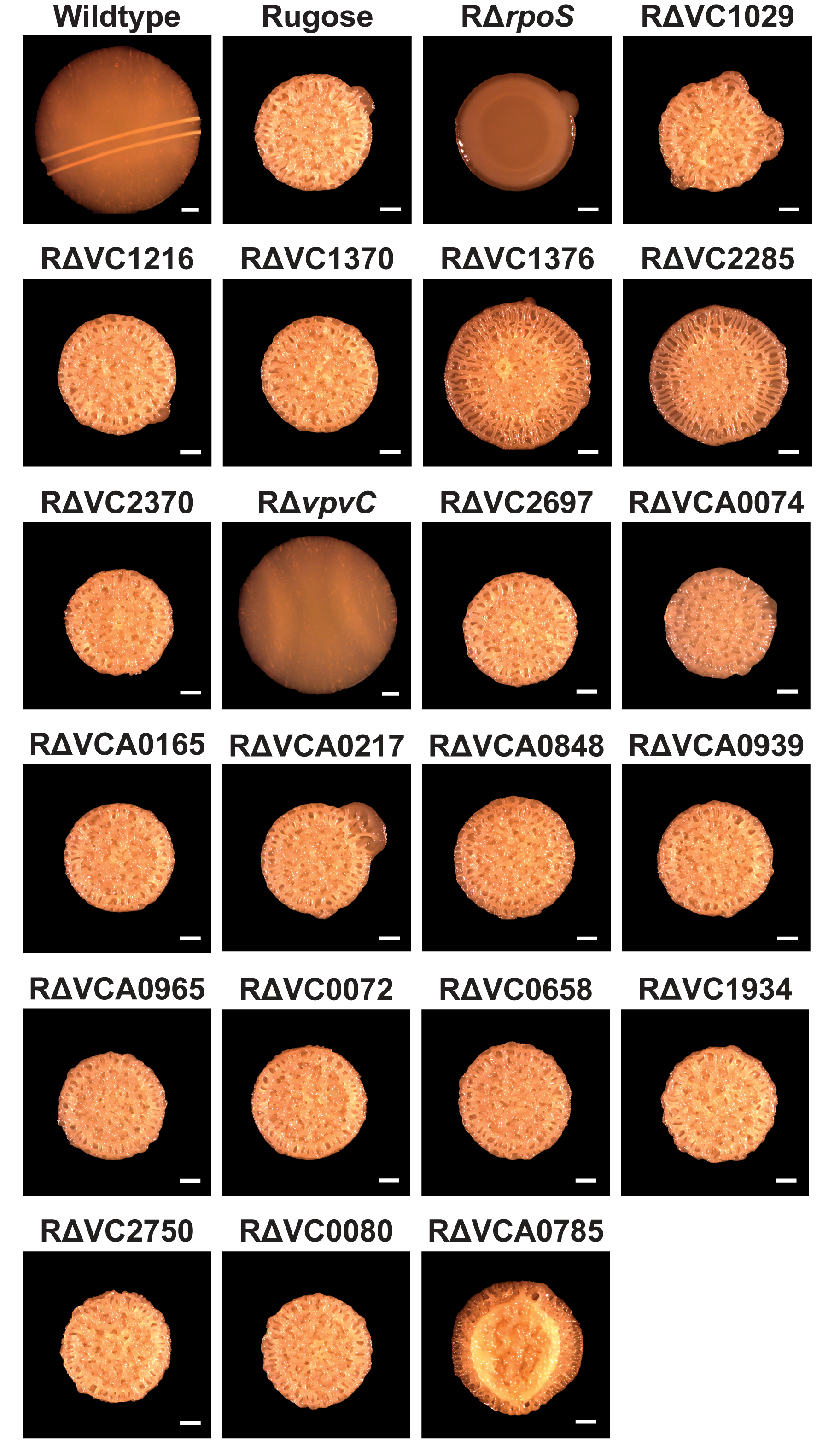

Supplement: S3 Fig — Representative images of deletion mutants of RpoS regulated DGCs in the rugose background. Colonies were grown for 72 hours at 30°C. (TIF) [file pgen.1011870.s005.tif]

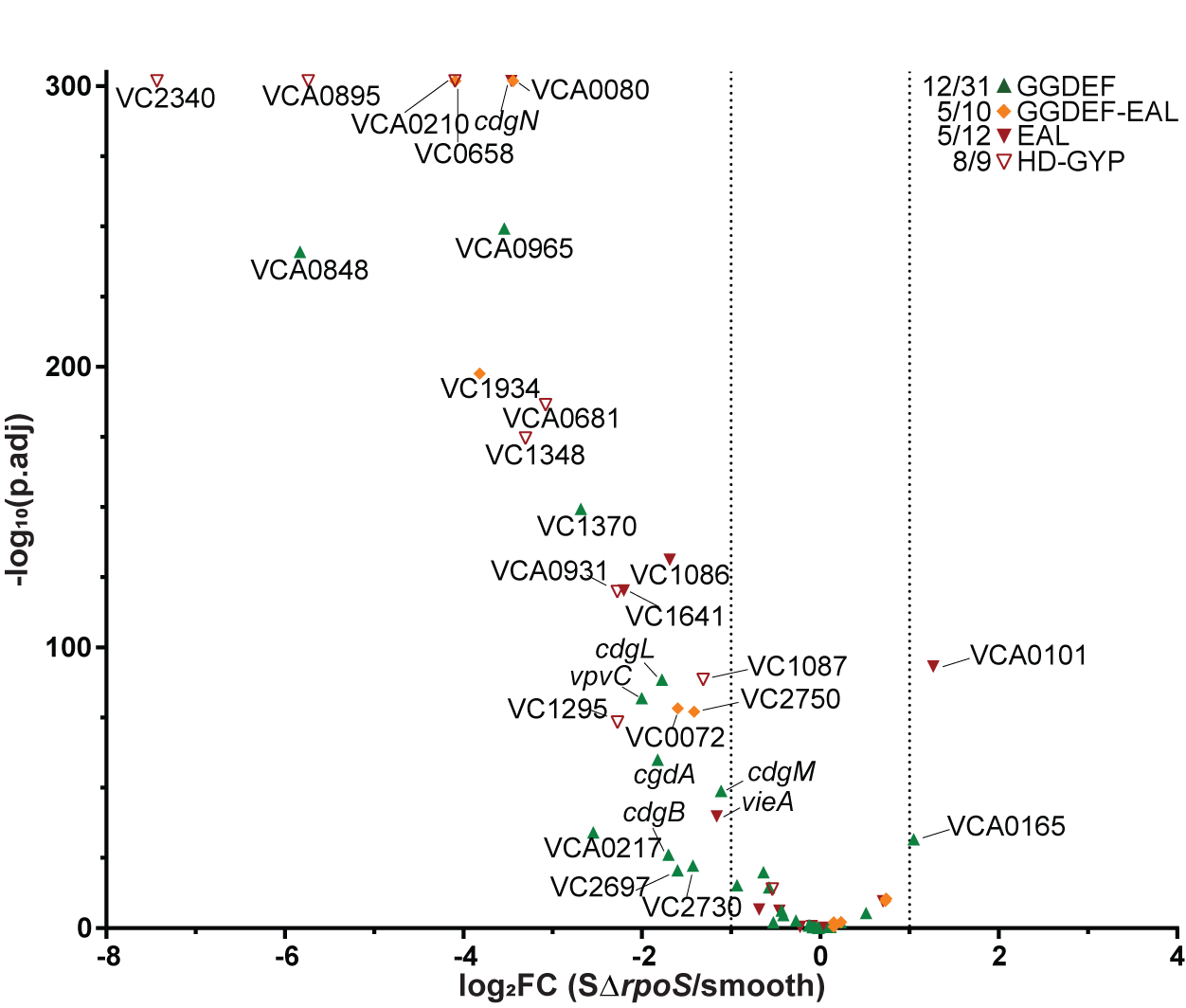

Supplement: S4 Fig — Volcano plot of differentially regulated c-di-GMP metabolism genes in ΔrpoS compared to the wild-type. Genes with GGDEF domains are represented by green upward triangles, GGDEF-EAL domains by orange diamonds, EAL domains by red downward triangles, and HD-GYP domains by red empty downward triangles. Dotted lines indicate a log2FC threshold of -1 and 1. (TIF) [file pgen.1011870.s006.tif]

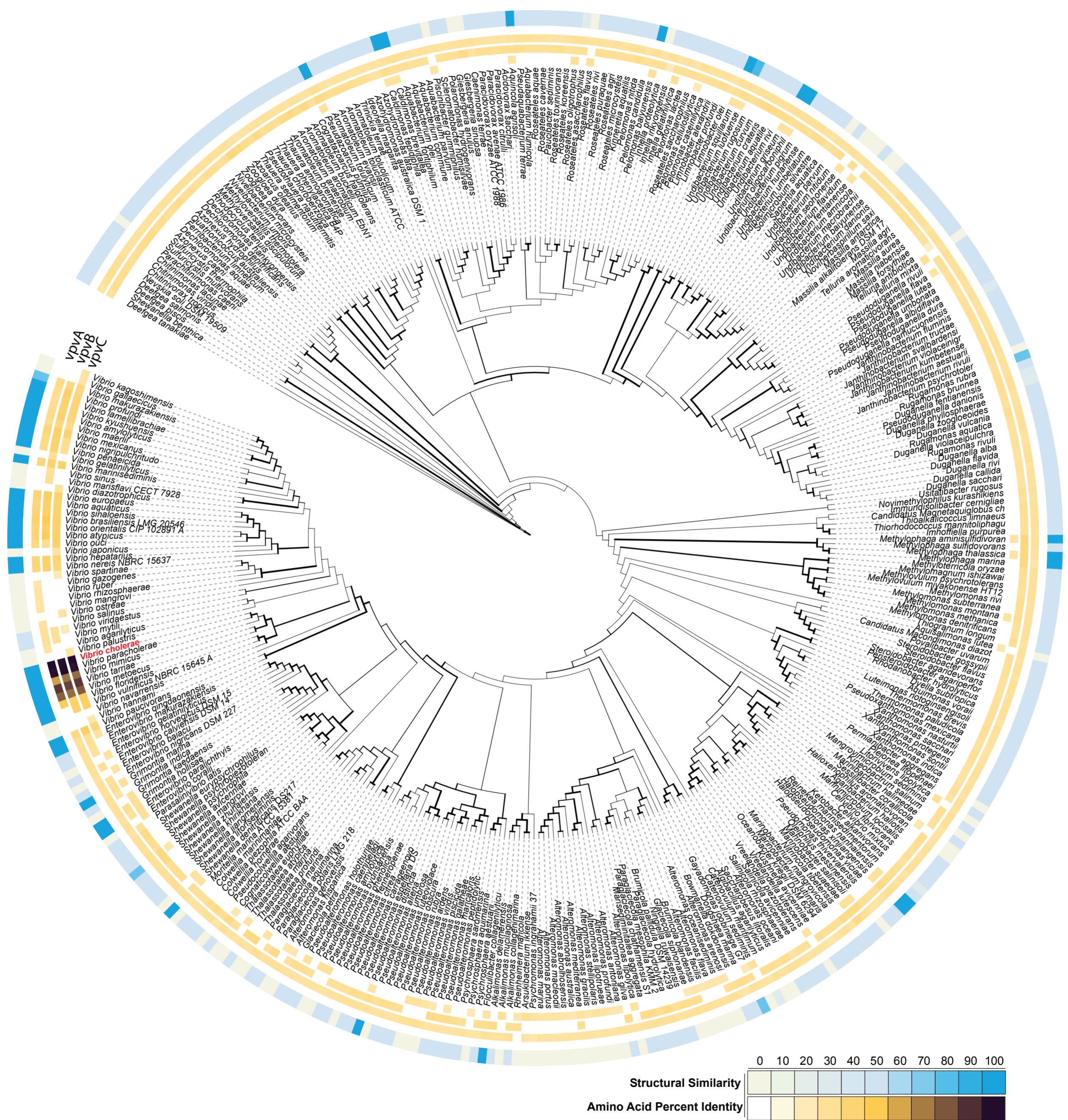

Supplement: S5 Fig — Conservation of the vpvABC operon across the Pseudomonadota, annotated in the context of a RecA phylogeny. All Vibrio reference genomes and all genomes with an average percent identity above 15% for vpv operon genes are represented. The percent amino acid conservation for the homologs of each Vpv operon component and the structural similarity for the entire operon (i.e., gene synteny) are presented as colored gradients. (PDF) [file pgen.1011870.s007.pdf]
